# Comparative Safety and Effectiveness of Ticagrelor versus Clopidogrel in Patients With Acute Coronary Syndrome: An On-Treatment Analysis From a Multicenter Registry

**DOI:** 10.3389/fcvm.2022.887748

**Published:** 2022-05-27

**Authors:** Manuel Almendro-Delia, Emilia Blanco-Ponce, Jesús Carmona-Carmona, J. A. Arboleda Sánchez, Juan Carlos Rodríguez Yáñez, José Manuel Soto Blanco, Isabel Fernández García, José M. Castillo Caballero, Juan C. García-Rubira, Rafael J. Hidalgo-Urbano

**Affiliations:** ^1^Acute Cardiovascular Care Unit, Hospital Universitario Virgen Macarena, Seville, Spain; ^2^Intensive Care Unit, Hospital Regional Universitario de Malaga, Malaga, Spain; ^3^Intensive Care Unit, Hospital de Puerto Real, Cadiz, Spain; ^4^Intensive Care Unit, Hospital San Cecilio, Granada, Spain; ^5^Intensive Care Unit, Hospital Costa del Sol, Malaga, Spain; ^6^Intensive Care Unit, Hospital Universitario Virgen de la Victoria, Malaga, Spain

**Keywords:** acute coronary syndrome, dual antiplatelet therapy, P2Y 12 inhibitor, exposure misclassification, medication adherence

## Abstract

**Background:**

The net clinical benefit of ticagrelor over clopidogrel in acute coronary syndrome (ACS) has recently been questioned by observational studies which did not account for time-dependent confounders. We aimed to assess the comparative safety and effectiveness of ticagrelor vs. clopidogrel accounting for non-adherence in a real-life setting.

**Methods:**

This is a prospective, multicenter cohort study of patients with ACS discharged on ticagrelor or clopidogrel between 2015 and 2019. Major exclusions were previous intracranial bleeding, and the use of prasugrel or oral anticoagulation. Association of P2Y_12_ inhibitor therapy with 1-year risk of Bleeding Academic Research Consortium Type 3 or 5 bleeding; major adverse cardiac events (MACEs), a composite endpoint of all-cause death, nonfatal myocardial infarction (MI), nonfatal stroke, or urgent target lesion revascularization; definite/probable stent thrombosis; vascular death; and net adverse clinical event (a composite endpoint of major bleeding and MACE) were analyzed according to the “on-treatment” principle, using fully adjusted Cox and Fine-Gray regression models with doubly robust inverse probability of censoring weighted estimators.

**Results:**

Among 2,070 patients (mean age 63 years, 27% women, 62.5% ST-elevation MI), 1,035 were discharged on ticagrelor and clopidogrel, respectively. Ticagrelor-treated patients were younger and had few comorbidities, but high rates of medication non-compliance, compared with clopidogrel users. After comprehensive multivariate adjustments, ticagrelor did not increase the risk of major bleeding compared with clopidogrel [subhazard ratio, 1.40; 95% confidence interval (CI), 0.96–2.05], while proved superior in reducing MACE (hazard ratio 0.62; 95% CI, 0.43–0.90), vascular death (subhazard ratio, 0.71; 95% CI, 0.52–0.97) and definite/probable stent thrombosis (subhazard ratio, 0.54; 95% CI, 0.30-0.79); thereby resulting in a favorable net clinical benefit (hazard ratio 0.78; 95% CI, 0.60–0.98) compared with clopidogrel. Results from sensitivity analyses were consistent with those from the primary analysis, whereas those from the intention-to-treat (ITT) analysis went in the opposite direction.

**Conclusion:**

Among all-comers with ACS, ticagrelor did not significantly increase the risk of major bleeding, while resulting in a net clinical benefit compared with clopidogrel. Further research is warranted to confirm these findings in high bleeding risk populations.

**CREA-ARIAM Andalucía:**

(ClinicalTrials.gov Identifier: NCT02500290); Current pre-specified analysis (ClinicalTrials.gov Identifier: NCT04630288).

## Graphical Abstract

Forest plot depicting the 1-year adjusted risk for the primary and secondary outcomes of ticagrelor versus clopidogrel in patients with acute coronary syndrome in the multicenter CREA-ARIAM Andalucía registry. Boldface font indicates statistical significance at P < 0.05. *The vertical dashed line in red set at 2.1 represents the estimated threshold for the upper bound of the two-sided 95% CI of the aSHR to reject the null hypothesis that clopidogrel is safer than ticagrelor in terms of relative risk for major bleeding. ACS, acute coronary syndrome; aHR, adjusted hazard ratio; aSHR, adjusted subhazard ratio; BARC, Bleeding Academic Research Consortium; CI, confidence interval; CREA, CRuce Entre Antiplaquetarios (Safety and Effectiveness of Switching Between Antiplatlets Agents in ACS); MACE, major adverse cardiovascular events; NACE, net adverse clinical events. 
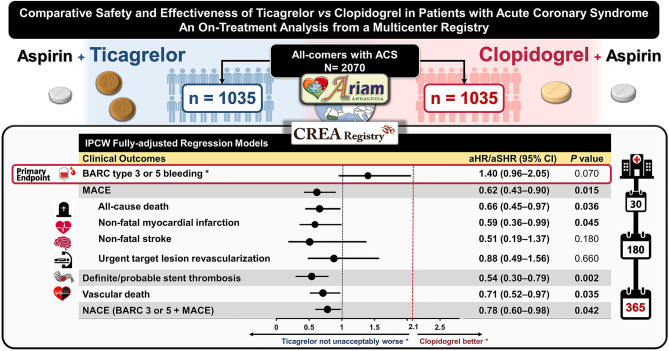

## Introduction

Dual antiplatelet therapy (DAPT) combining aspirin and a P2Y_12_ receptor inhibitor is the mainstay of treatment and secondary prevention after acute coronary syndrome (ACS) (1, 2). In the PLATelet inhibition and patient Outcomes (PLATO) trial, ticagrelor provided greater reductions in major ischemic events, with an acceptable safety profile compared with clopidogrel (3, 4). These findings have been confirmed in real-life settings (5, 6). Based on this, current guidelines on DAPT from the American College of Cardiology/American Heart Association and European Society of Cardiology, ticagrelor over clopidogrel in ACS is recommended regardless of the intended management, unless contraindicated or high bleeding risk (1, 2).

Despite these recommendations, an increasing body of evidence has recently shown that ticagrelor is not significantly associated with a reduction in ischemic events, but with an increased risk of major bleeding compared with clopidogrel (7–13). These seemingly conflicting results have raised concerns over the actual net clinical benefit of ticagrelor in nontrial settings. In this context, drug-related side effects, such as dyspnea or bleeding may warrant premature discontinuation of ticagrelor or switch to a less potent agent, which could limit any potential advantage of ticagrelor over clopidogrel in daily practice (14). Nevertheless, not much research has gone into the methods of accounting for non-adherence of medication in estimating the safety and effectiveness of P2Y_12_ inhibitors in real-world settings. Therefore, given the common occurrence of de-escalation strategies from ticagrelor to clopidogrel in daily practice (14), and that the overwhelming majority of previous studies relied on the intention-to-treat (ITT) principle; interpreting and understanding the generalizability of the existing evidence can be challenging due to the potential misclassification bias introduced by the ITT analysis. In the light of this uncertainty, we aimed to assess the safety and effectiveness of ticagrelor vs. clopidogrel among patients with ACS, accounting for the non-adherence to medication in a contemporary real-life setting.

## Methods

### Study Design and Population

This is a pre-specified analysis (NCT04630288) from the CREA (Safety and Effectiveness of Switching Between Antiplatelet Agents, NCT02500290) study; a prospective, multicenter investigator-initiated branch of the main ARIAM-Andalucía (Analysis of Delay in Acute Myocardial Infarction in Andalucía) registry. The CREA study aimed at assessing the prevalence and prognostic impact of nonadherence with P2Y_12_ inhibitors after ACS. Details of design and primary results from the ARIAM-Andalucía registry have already been reported elsewhere (15, 16). Briefly, established in 1994, ARIAM-Andalucía is an all-comer, prospective, multicenter real-world registry of consecutive patients with ACS admitted to cardiac intensive care units in the Autonomous Community of Andalucía (Southern Spain). The current analysis was conducted in six teaching hospitals with percutaneous coronary intervention (PCI) and coronary artery bypass grafting (CABG) surgery facilities. Patients aged 18 years or older discharged alive after an ACS-related hospitalization between April 2015 and April 2019, were prospectively screened for eligibility if they were intended to receive clopidogrel or ticagrelor on top of aspirin, as part of DAPT for at least 12 months following the index ACS. Major exclusions were the use of prasugrel or chronic oral anticoagulation, a recent major bleeding or a previous intracranial hemorrhage at any time, and patients lost to follow-up or with missing data (more details in Supplementary Methods 1). The study conforms to the provisions of the Declaration of Helsinki and follows the Strengthening the Reporting of Observational Studies in Epidemiology (STROBE) Statement. The Regional Research Ethics Committee of Andalusia and the Institutional Review Boards at each participating center approved this study. All eligible patients were required to give written informed consent before participating.

### Definition of Outcomes

The primary outcome was major bleeding defined as Bleeding Academic Research Consortium (BARC) Type 3 or 5 bleeding (17). The main secondary endpoint was a major adverse cardiac event (MACE), a composite endpoint defined as the first occurrence of all-cause death, nonfatal myocardial infarction (MI) (18), nonfatal stroke, or an unplanned/urgent target lesion revascularization. Further secondary outcomes included death from vascular causes (including cardiac and cerebrovascular mortality), definite/probable stent thrombosis according to the Academic Research Consortium criteria (19), and net adverse clinical event (a composite endpoint of major bleeding and MACE) (additional information in Supplementary Methods 2).

### Exposure and Outcome Ascertainment

Clinical outcomes were prospectively tracked within 1 year after the index ACS by structured telephone interviews with patients or relatives and during scheduled post-discharge or outpatient follow-up visits. Self-reported information from patients was validated with data manually extracted from electronic medical records. All suspected clinical events identified during the follow-up underwent formal blinded adjudication by consensus of two experienced investigators, who were blinded—to calendar year and exposure status—using previously anonymized original source data. Discrepancies were resolved through a consensus discussion with a third consultant cardiologist not involved in the study (Details on the endpoint adjudication process are presented in Supplementary Methods 2). Adherence was measured at time intervals by the medication possession ratio (MPR) metric (20), using data from the Andalusian Electronic Drug Prescription and Dispensation Registry (refer to Exposure Ascertainment in Supplementary Methods 2). Treatment switching (escalation/de-escalation) was identified by the discontinuation of the initial P2Y_12_ inhibitor and initiation of the alternative medication. The exposure status was prospectively ascertained as a time-dependent variable according to the “on-treatment” principle (i.e., treatment actually received). In order to deal with informative censoring, we applied different censoring schemes to define the “at-risk” time window for hemorrhagic and ischemic outcomes separately (21). Therefore, for bleeding events, the “on-treatment” period was defined as the time from the index admission date until the end of persistent drug exposure plus a 14-day “washout” period (offset of platelet inhibition) (22). On the other hand, since the accrued risk of ischemic events associated with DAPT discontinuation may persist for some time after the treatment was stopped (time-lag effect) (23), a lag-censoring approach was implemented by applying a 90-day lag period for censoring after treatment discontinuation (24) (Supplementary Methods 2). The last follow-up date was April 30, 2020.

### Statistical Analysis

#### Sample Size Estimation

The primary hypothesis was that the safety profile of ticagrelor is not unacceptably worse than that of clopidogrel, assuming an expected annual major bleeding rate of 5.6% for both groups (11). Therefore, assuming a type I error of 5% with a two-sided 95% confidence interval (CI)—equivalent to a one-sided 97.5% CI with a 2.5% of the level of significance—after adjusting for an anticipated switching rate of 9%, and 13% follow-up loss, a sample size of 1,900 subjects would provide at least 80% of power to detect the upper bound limit of the two-sided 95% of CI for the adjusted subhazard ratio (aSHR) of major bleeding for ticagrelor vs. clopidogrel not to exceed 2.10. This margin was selected on the basis of the existing literature (11) and represents the clinically acceptable excess risk of bleeding that would be expected for ticagrelor relative to clopidogrel in a real-world setting (Supplementary Methods 2).

#### Statistical Modeling

For descriptive analysis, patients were grouped according to the P2Y_12_ inhibitor prescribed at hospital discharge. Categorical variables are presented as frequencies and percentages and were compared using the χ2 test or the Fisher's test as appropriate. Continuous variables are reported as mean and standard deviation (SD), or as median and interquartile range (IQR) and were compared using independent samples from Student's *t*-tests and Mann–Whitney U tests, as appropriate. The inverse probability of censoring weighting (IPCW) approach was used to account for potential informative censoring resulting from time-varying predictors, such as the differential non-adherence to P2Y_12_ inhibitors observed in daily practice (25). In brief, the IPCW method is a time-varying exposure analysis, whereby patients are artificially censored at the time of switching or discontinuing therapies. Thereafter, to adjust for the potential selection bias induced by this artificial censoring, the remaining observations are weighted, which are inversely proportional to the estimated probability of remaining uncensored (i.e., the remaining of the initial P2Y_12_ inhibitor) up to the end of the follow-up, conditional on the baseline and time-varying predictors (Supplementary Methods 2). Incidences of outcomes are summarized using weighted Kaplan-Meier estimators or cumulative incidence functions, as appropriate. The association between the exposure and outcomes was evaluated according to the “on-treatment principle,” taking death as a competing event. To this end, fully adjusted Cox proportional hazards regression and Fine–Gray competing risks regression models were fitted including doubly robust IPCW estimators, with participating hospitals entered as a random-effect variable (26) (Supplementary Methods 2). Results were expressed as adjusted hazard ratios (aHRs) or aSHRs with their 95% CIs. A set of sensitivity analyses were conducted to ensure the robustness of the primary analysis (Supplementary Methods 2). These included subgroup analysis and a 30-day landmark analysis for the primary outcome. In addition, propensity score matching (PSM) (27) and instrumental variable (IV) analysis using the calendar year as the instrument (28), were used to adjust for selection bias and unmeasured bias, respectively. Finally, a conventional ITT analysis, which only considered baseline exposures assuming full adherence (time-fixed analysis), was performed to better enable comparison with previous studies. All analysis tests were two-tailed with alpha set at 5%. All analyses were performed using Stata 13.1 (StataCorp. 2013. Stata Statistical Software: College Station, TX: StataCorp LP).

## Results

### Patient Characteristics

**Figure 1:**
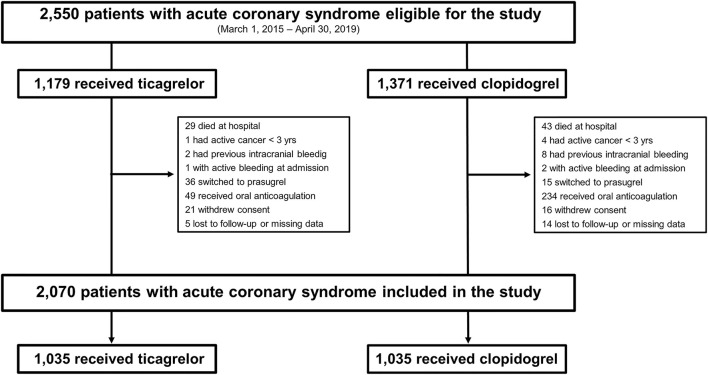
Flow chart of patients.

Out of 2,550 patients screened for eligibility, 2,070 fulfilled the inclusion criteria, of whom 1,035 patients were discharged on ticagrelor and clopidogrel, respectively (Figure 1). The use of ticagrelor increased significantly over the study period (Supplementary Figure S1). Tables 1, 2 summarize the baseline and procedural characteristics of patients according to the P2Y_12_ inhibitor prescribed at hospital discharge. Overall, ticagrelor-treated patients were younger and less likely to be women, had less comorbidity, were more likely to undergo PCI with drug-eluting stents (DES) than CABG surgery as the preferred revascularization choice, and were also more likely to receive evidence-based therapies at discharge, compared with clopidogrel users.

**Table 1:** Baseline characteristics of patients according to the P2Y_12_ inhibitor prescribed at hospital discharge.

	**Overall Cohort**	**Ticagrelor**	**Clopidogrel**	***P* value**
	**(n = 2070)**	**(n = 1035)**	**(n = 1035)**	
Age, years	63 (54–73)	61 (53–70)	66 (56–76)	<0.001
≥ 75 years	456 (22.0)	159 (15.5)	297 (28.5)	<0.001
Sex, Male	1517 (73.0)	802 (77.5)	715 (70.0)	<0.001
Body mass index, kg/m^2^	27.7 (4.0)	28.5 (4.1)	27.3 (4.3)	<0.001
**Medical history**				
Current smoker	866 (42.0)	481 (46.5)	385 (37.2)	<0.001
Hypertension	1166 (56.0)	535 (51.7)	631 (61.0)	<0.001
Diabetes mellitus	643 (31.0)	288 (28.0)	355 (34.0)	0.001
Hyperlipidaemia	937 (45.0)	463 (44.7)	474 (46.0)	0.62
Peripheral arterial disease	115 (5.5)	39 (4.0)	76 (7.3)	<0.001
Chronic obstructive pulmonary disease	118 (5.7)	48 (4.6)	70 (6.8)	0.04
Chronic kidney disease	135 (6.5)	47 (4.5)	88 (8.5)	<0.001
Dialysis	36 (1.7)	10 (1.0)	26 (2.5)	0.007
History of atrial fibrillation	43 (2.3)	17 (1.6)	30 (2.9)	0.06
Myocardial infarction	304 (14.7)	134 (13.0)	170 (16.4)	0.02
Percutaneous coronary intervention	302 (14.5)	135 (13.0)	167 (16.0)	0.04
Coronary artery bypass grafting	39 (2.0)	17 (1.6)	22 (2.0)	0.41
Stroke	153 (7.5)	46 (4.4)	107 (10.2)	0.03
Previous bleeding	58 (3.0)	12 (1.2)	46 (4.4)	<0.001
Anemia	74 (3.5)	24 (2.4)	50 (4.8)	0.002
Cancer *	39 (1.9)	14 (1.4)	25 (2.4)	0.08
**Clinical presentation**				
Non-ST-segment elevation ACS	779 (36.5)	334 (32.5)	445 (43.0)	<0.001
Non-ST-segment elevation MI	686 (33.0)	306 (29.5)	380 (36.5)	
Unstable angina	93 (4.5)	28 (2.5)	65 (6.5)	
ST-segment elevation MI	1291 (62.5)	701 (67.5)	590 (57.0)	<0.001
Killip class ≥ 2	255 (12.3)	107 (10.3)	148 (14.0)	0.006
CRUSADE score	25 (14–38)	20 (11–32)	29 (19–43)	<0.001
GRACE score	136 (113–161)	133 (112–156)	140 (115–167)	<0.001
Creatinine clearance, ml/min/1.73 m^2^	85 (60–110)	94 (69–120)	76 (54–99)	<0.001
Left ventricular ejection fraction, %	52.0 (11)	52.0 (10.4)	52.4 (11.3)	0.82

*Data summarized as mean (SD), median (IQR) and n (%).*

* *Diagnosis of cancer more than 3 years before the index ACS.*

*ACS, acute coronary syndrome; CRUSADE, Can Rapid risk stratification of Unstable angina patients Suppress ADverse outcomes with Early implementation of the ACC/AHA guidelines; GRACE, Global Registry of Acute Coronary Events; MI, myocardial infarction*.

**Table 2:** Procedural characteristics and medication at discharge.

	**Overall Cohort (n = 2070)**	**Ticagrelor** **(n = 1035)**	**Clopidogrel (n = 1035)**	***P*-value**
**Procedural characteristics**				
Radial artery approach	1363 (66.0)	710 (68.5)	653 (63.0)	0.003
Multivessel disease*	931 (45.0)	472 (45.5)	459 (44.3)	0.56
Chronic total occlusion	186 (9.0)	73 (7.0)	113 (11.0)	0.002
Complete revascularization ^†^	537 (57.7)	288 (61.0)	249 (54.3)	0.03
Glycoprotein IIb/IIIa inhibitors	230 (11.0)	127 (12.0)	103 (10.0)	0.09
Reperfusion therapy, STEMI	**n** **=** **1291**	**n** **=** **701**	**n** **=** **590**	0.05
Primary PCI	1082 (84.0)	604 (86.0)	478 (81.0)	
Pharmacoinvasive strategy ^‡^	209 (16.0)	97 (14.0)	112 (18.5)	
Management strategy, NSTE-ACS	**n** **=** **779**	**n** **=** **334**	**n** **=** **445**	0.09
Conservative	11 (0.5)	2 (0.5)	9 (2.0)	
Invasive	768 (98.5)	332 (99.5)	436 (98.0)	
Reperfusion strategy, all-comers				<0.001
PCI, any	1857 (89.7)	1009 (97.5)	848 (82.0)	
Drug-eluting stent	1608 (86.5)	918 (93.0)	690 (85.3)	
CABG surgery	41 (2.0)	2 (0.2)	39 (3.8)	
Medical treatment	172 (8.4) 4)	24 (2.3)	148 (14.3)	
**Medication at discharge/Adherence**				
β-Blocker	1789 (86.4)	903 (87.0)	886 (85.6)	0.27
Statin	2000 (96.6)	1018 (98.4)	982 (95.2)	<0.001
RAAS blocker	1849 (89.3)	962 (93.0)	887 (86.0)	<0.001
Proton-pump inhibitor	1591 (77.0)	833 (80.5)	758 (73.3)	<0.001
P2Y_12_ inhibitor switching ^§^	102 (4.9)	79 (7.6)	23 (2.2)	<0.001
Time-to-switch, days	47.5 (7–148)	45 (6–131)	93 (12–171)	0.27
Duration of DAPT, days	365 (49)	369 (36)	360 (60)	0.06
Medication possession ratio (1 year), %	70 (21)	68 (31)	71 (29)	0.63

*Data summarized as mean (SD), median (IQR), and n (%).*

* *Patients with multivessel disease, defined as at least two major vessels (≥ 2 mm diameter) from a different territory with lesions deemed angiographically significant (≥50% stenosis of the left main stem, ≥70% stenosis in other major coronary vessel, or 30% to 70% stenosis with fractional flow reserve ≤ 0.8).*

† *For patients with multivessel disease.*

‡ *Ninety-seven ticagrelor users in the pharmacoinvasive group switched from clopidogrel at least 24 h after receiving fibrinolytic therapy for STEMI.*

§ *After hospital discharge.*

*ACS, acute coronary syndrome; CABG, coronary artery bypass grafting; DAPT, dual antiplatelet therapy; NSTE-ACS, Non-ST-segment elevation; PCI, percutaneous coronary intervention; RAAS, renin-angiotensin-aldosterone system; STEMI, ST-segment elevation myocardial infarction.*

*Boldface font indicates the total number of subjects in each category*.

### Medication Adherence

After 1 year, significantly more clopidogrel than ticagrelor users prematurely discontinued the assigned treatment at the discretion of the physician Supplementary Tables S1, S2), whereas cessation of DAPT due to noncompliance was more common among the latter group (Supplementary Table S3). Drug switching occurred in 5% of the patients during the follow-up period, mostly (*n* = 45, 44%) within 30 days after discharge, and particularly among patients initially treated with ticagrelor (Table 2). The most common reason for de-escalation from ticagrelor to clopidogrel was dyspnea followed by bleeding, while the occurrence of thrombotic events was the most frequent cause of switching from clopidogrel to ticagrelor (Table 3).

**Table 3:** Reasons for and timing of P2Y_12_ inhibitor switching.

**Modality and indication for switching**	**N (%)**	**Time to switch, days**
**Total number of switchers**	**102 (4.9)**	**47.5 (7.0–148.0)**
**Ticagrelor to clopidogrel (“de–escalation”)**	**79 (7.6)**	**45.0 (6.0–131.0)**
Dyspnea	25 (31.6)	30.7 (12.2–132.2)
Bleeding event	13 (16.5)	51.0 (12.0–97.6)
Physician's decision	12 (15.2)	93.0 (55.0–188.0)
Economic reason	10 (12.7)	3.5 (2.0–7.2)
Recurrent ischemic event (stroke)	4 (5.0)	126.4 (33.2–219.0)
Adverse effect, other	3 (3.8)	45.3 (21.7–203.0)
Need for invasive procedure	3 (3.5)	28.7 (10.0–297.0)
Cardiac pauses	2 (2.5)	9.7 (1.6–9.7)
High bleeding risk	2 (2.5)	4.8 (4.1–4.8)
Contraindication	2 (2.5)	3.3 (2.3–3.3)
Low adherence	1 (1.3)	101.0
Poor compliance to twice–daily dosing	1 (1.3)	26.0
**Clopidogrel to ticagrelor (“escalation”)**	**23 (2.2)**	**93.0 (12–171)**
Recurrent ischemic event (MI, ST)	13 (56.5)	110.0 (11.0–197.7)
Physician's decision	6 (26.0)	73.0 (9.0–178.0)
High thrombotic burden	4 (17.5)	23.7 (13.0–46.0)

*Values expressed as n (%), median (IQR).*

*IQR, interquartile range; MI, myocardial infarction; ST, stent thrombosis. Boldface font indicates the total number of subjects and timing of switching in each category*.

### Clinical Outcomes

**Figure 2:**
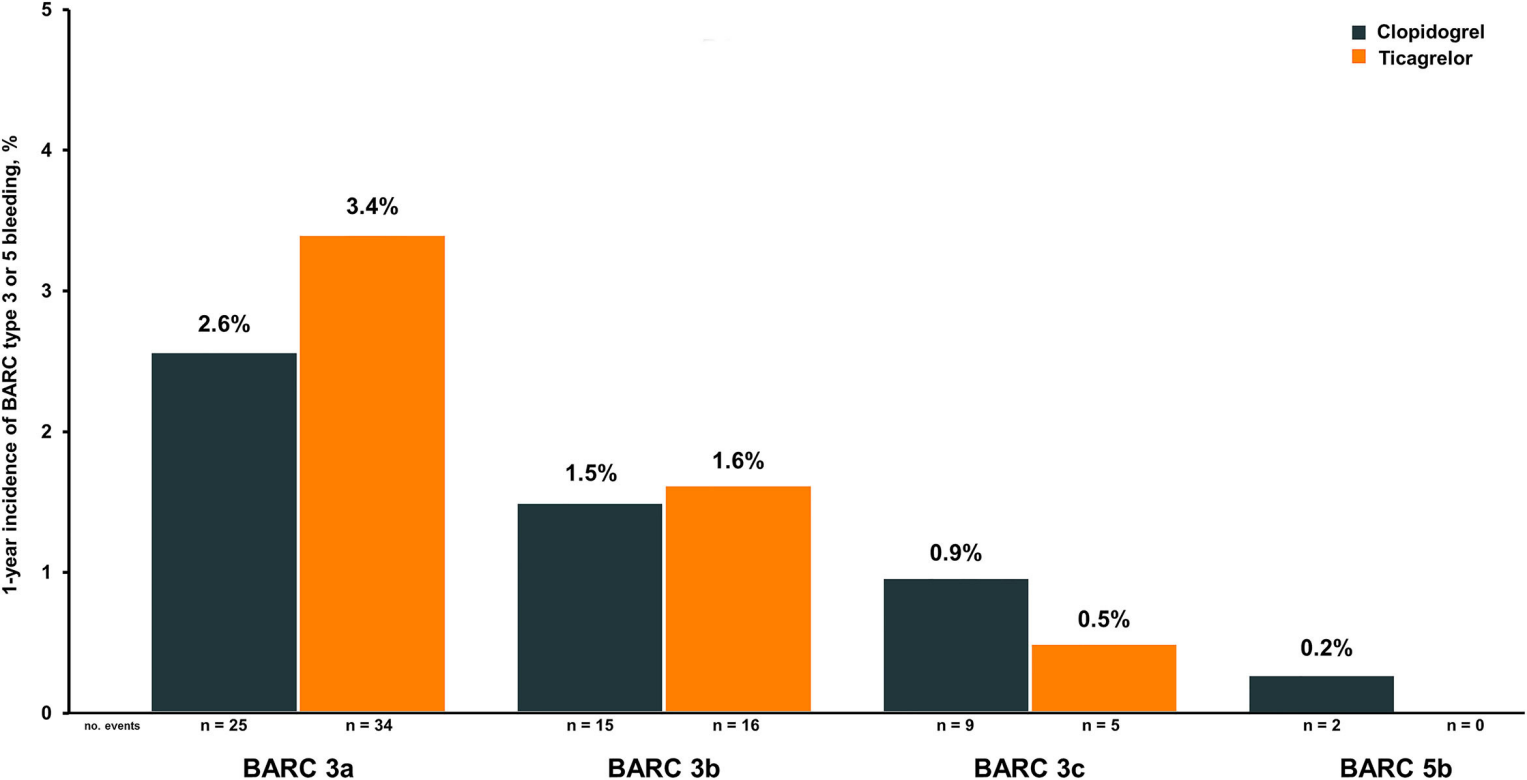
Cumulative incidence of BARC Type 3 and Type 5 bleeding events in patients treated with ticagrelor and clopidogrel. BARC, Bleeding Academic Research Consortium.

The 1-year cumulative incidence for the primary and secondary outcomes is listed in Table 4. A total of 106 major bleeding events occurred during 1,871.4 person-years of follow-up. Overall, bleeding complications occurred more frequently with ticagrelor than with clopidogrel (Figure 2). However, after comprehensive multivariate adjustment, the risk of major bleeding was not significantly superior compared to ticagrelor vs. clopidogrel, with the upper bound of the 95% CI for the relative risk of major bleeding of ticagrelor vs. clopidogrel found below the pre-specified margin of 2.10 (aSHR 1.40; 95% CI, 0.96–2.05). After one year, the MACE was found to have occurred in 198 (10.2%) patients (1,949.5 total person-years at risk). Exploratory analysis revealed that ticagrelor significantly reduced the incidence of MACE, definite/probable stent thrombosis, and vascular death, thereby resulting in a favorable net clinical benefit compared with clopidogrel (Table 4).

**Table 4:** Association between the primary and secondary outcomes and use of ticagrelor vs. clopidogrel.

	**Cumulative incidence**, ***no. events*** **(%)** *			**Unadjusted Models** ^**†**^		**Full adjusted Models** ^**‡**^	
	**Total**	**Ticagrelor**	**Clopidogrel**	**HR/SHR**	***P***	**aHR/aSHR**	***P***
	**(*n*= 2070)**	**(*n* = 1035)**	**(*n* = 1035)**	**(95% CI)**	**value**	**(95% CI)**	**value**
**Primary Outcome**							
**BARC type 3 or 5 bleeding**	106 (5.3)	55 (5.6)	51 (5.2)	1.07 (0.71–1.62)	0.730	1.40 (0.96–2.05)	0.070
**Secondary Outcomes**							
**MACE**	198 (10.2)	63 (6.3)	135 (13.7)	0.48 (0.29–0.79)	**0.004**	0.62 (0.43–0.90)	**0.010**
All–cause death	105 (5.0)	30 (2.9)	75 (7.1)	0.40 (0.24–0.68)	**0.001**	0.66 (0.45–0.97)	**0.030**
Non–fatal MI	82 (4.2)	24 (2.5)	58 (5.8)	0.43 (0.24–0.78)	**0.006**	0.59 (0.36–0.99)	**0.040**
Non–fatal stroke	23 (1.1)	7 (0.7)	16 (1.6)	0.42 (0.17–1.04)	0.060	0.51 (0.19–1.37)	0.180
uTLR	66 (3.3)	29 (3.0)	37 (3.6)	0.82 (0.41–1.65)	0.590	0.88 (0.49–1.56)	0.660
**Stent thrombosis** ^§^	59 (2.9)	21 (2.1)	38 (3.7)	0.56 (0.37–0.86)	**0.008**	0.54 (0.30–0.79)	**0.002**
**Vascular death**	55 (2.7)	19 (1.8)	36 (3.5)	0.53 (0.37–0.77)	**0.001**	0.71 (0.52–0.97)	**0.030**
**NACE**	274 (13.3)	108 (10.6)	166 (15.9)	0.62 (0.41–0.92)	**0.002**	0.78 (0.60–0.98)	**0.040**

*Boldface font indicates statistical significance at P <0.05.*

* *(%)are weighted Kaplan–Meier estimates or cumulative incidence functions at 1 year, as appropriate.*

† *Univariate IPCW Cox and Fine–Gray regression models, with hospitals entered as a random–effects variable (cluster robust).*

‡ *Fully adjusted IPCW Cox and Fine–Gray regression models with robust variance estimators.*

§ *Definite or probable.*

*ACS, acute coronary syndrome; aHR, adjusted hazard ratio; aSHR, adjusted subhazard ratio; BARC, Bleeding Academic Research Consortium; CI, confidence interval; MACE, major adverse cardiovascular events; MI, myocardial infarction; NACE, net adverse clinical events; uTLR, urgent target lesion revascularization*.

### Sensitivity Analysis

The result from the primary outcome was consistent across most subgroups, although there was a signal of an increased risk of bleeding among elderly patients and those with a history of bleeding exposed to ticagrelor (*P*_interaction_ = 0.05, and 0.04, respectively), compared with their counterparts treated with clopidogrel (Supplementary Figure S2). A 30-day landmark analysis was performed to account for procedural-related bleedings during hospitalization and poor adherence with P2Y_12_ inhibitors within the first 30 days from index admission. The risk of BARC Type 3 or 5 bleeding was higher during the acute period, and was subsequently decreased during the follow-up. However, neither the adjusted risk of major bleeding within the first 30 days post-admission nor from this time point onwards was found to be different comparing ticagrelor vs. clopidogrel (Supplementary Figure S3). After PSM analysis, using a fine-tuned selection of calipers, a sample of 801 well-balanced pairs with ticagrelor and clopidogrel was obtained, representing almost 80% of the total cohort (Table 5, Supplementary Figure S4). The results from the PS-matched sample went in the same direction as those from the primary analysis (Supplementary Table S4). Likewise, after accounting for unmeasured confounding, findings from the IV analysis were also consistent with those of the primary analysis (Supplementary Tables S5–S7). Conversely, the results from ITT analysis and those from primary analysis went in opposite directions, with the former showing no significant reductions in MACE, but a significant increase in bleeding risk with ticagrelor vs. clopidogrel, both in the overall and in the PSM sample (Supplementary Table S8).

**Table 5:** Standardized covariate mean differences stratified by treatment before and after matching.

	**Before propensity score matching**			**After propensity score matching**		
	**Ticagrelor**	**Clopidogrel**	**Standardized**	**Ticagrelor**	**Clopidogrel**	**Standardized**
	**(n = 1035)**	**(n = 1035)**	**differences**	**(n = 801)**	**(n = 801)**	**differences**
Age, years	61 (53, 70)	66 (56, 76)	−0.45	64 (57, 73)	63 (55, 73)	−0.03
≥ 75 yrs	159 (15.5)	297 (28.5)	−0.32	157 (19.6)	175 (21.8)	−0.03
Sex, Male	802 (77.5)	715 (70.0)	0.12	581 (72.5)	592 (74.0)	0.02
Body mass index, kg/m^2^	28.5 (4.1)	27.3 (4.3)	0.27	27.6 (4.0)	27.4 (4.2)	0.05
**Medical history**						
Current smoker	481 (46.5)	385 (37.2)	0.19	331 (41.0)	344 (43.0)	−0.04
Hypertension	535 (51.7)	631 (61.0)	−0.19	447 (56.0)	447 (56.0)	0.00
Diabetes mellitus	288 (28.0)	355 (34.0)	−0.13	241 (30.0)	254 (31.5)	−0.03
Hyperlipidemia	463 (44.7)	474 (46.0)	−0.03	366 (45.7)	356 (44.0)	0.03
Peripheral arterial disease	39 (4.0)	76 (7.3)	−0.14	38 (5.0)	50 (6.0)	−0.04
Chronic obstructive pulmonary disease	48 (4.6)	70 (6.8)	−0.10	38 (4.7)	50 (6.0)	−0.06
Chronic kidney disease	47 (4.5)	88 (8.5)	−0.16	47 (6.0)	52 (6.5)	−0.02
Dialysis	10 (1.0)	26 (2.5)	−0.11	10 (1.2)	16 (2.0)	−0.06
Previous myocardial infarction	134 (13.0)	170 (16.4)	−0.10	119 (15.0)	119 (15.0)	0.00
Previous PCI	135 (13.0)	167 (16.0)	−0.09	117 (14.5)	117 (14.5)	0.00
Previous CABG	17 (1.6)	22 (2.0)	−0.03	16 (2.0)	16 (2.0)	0.00
History of stroke	46 (4.4)	107 (10.2)	−0.22	46 (5.7)	55 (6.8)	−0.05
History of heart failure (NYHA class > III)	12 (1.2)	25 (2.4)	−0.10	12 (1.5)	12 (1.5)	0.00
Previous major bleeding	12 (1.2)	46 (4.4)	−0.19	12 (1.5)	19 (2.3)	−0.06
History of anemia	24 (2.4)	50 (4.8)	−0.15	24 (3.0)	30 (3.7)	−0.04
Previous cancer *	14 (1.4)	25 (2.4)	−0.07	14 (1.9)	19 (2.3)	−0.03
**Clinical presentation**						
Non–ST–segment elevation ACS	334 (32.5)	445 (43.0)	−0.22	315 (39.2)	297 (37.3)	0.04
Non–ST–segment elevation MI	306 (29.5)	380 (36.5)		287 (35.7)	250 (31.5)	
ST–segment elevation MI	701 (67.5)	590 (57.0)	0.22	486 (60.8)	504 (62.7)	−0.04
Killip class ≥ 2	107 (10.3)	148 (14.0)	−0.11	106 (13.0)	86 (11.2)	0.06
CRUSADE score	20 (11, 32)	29 (19, 43)	−0.48	25 (17, 37)	25 (16, 37)	0.01
GRACE score	133 (112, 156)	140 (115, 167)	−0.17	141 (118, 165)	138 (111,157)	0.07
Creatinine clearance, ml/min/1.73 m^2^	94 (69, 120)	76 (54, 99)	0.39	83 (62, 103)	83 (60, 107)	0.01
LVEF at discharge, %	52.0 (10.4)	52.4 (11.3)	0.01	51.7 (10.5)	52.0 (11.0)	−0.02
**Procedural characteristics**						
Radial artery approach	710 (68.5)	653 (63.0)	0.13	535 (66.5)	505 (63.5)	0.06
Multivessel disease	472 (45.5)	459 (44.3)	0.02	362 (45.3)	343 (43.0)	0.04
Chronic total occlusion	73 (7.0)	113 (11.0)	−0.14	52 (6.5)	64 (8.0)	−0.06
Complete revascularization	288 (61.0)	249 (54.3)	0.14	221 (61.2)	207 (60.3)	0.02
Stent type, DES	918 (93.0)	690 (84.4)	0.28	677 (89.2)	573 (86.5)	0.08
Glycoprotein IIb/IIIa inhibitors	127 (12.0)	103 (10.0)	0.06	93 (11.5)	85 (10.6)	0.02
Reperfusion therapy, STEMI	**(n** **=** **701)**	**(n** **=** **590)**		**(n** **=** **486)**	**(n** **=** **504)**	
Primary PCI	604 (86.0)	478 (81.0)	0.14	398 (81.8)	412 (81.7)	0.00
Pharmacoinvasive strategy	97 (14.0)	112 (18.5)	−0.11	88 (18.2)	92 (18.2)	0.00
Management strategy, NSTE–ACS	**(n** **=** **334)**	**(n** **=** **445)**		**(n** **=** **315)**	**(n** **=** **297)**	
Invasive	332 (99.5)	436 (98.0)	0.14	313 (99.4)	293 (98.7)	0.07
Conservative	2 (0.5)	9 (2.0)	−0.13	2 (0.6)	4 (1.3)	−0.07
Reperfusion strategy, all–comers						
PCI, any	1009 (97.5)	848 (82.2)	0.52	777 (97.0)	768 (96.0)	0.04
CABG	2 (0.2)	39 (3.7)	−0.25	2 (0.2)	5 (0.6)	−0.06
Medical treatment	24 (2.3)	147 (14.2)	−0.56	22 (2.7)	28 (3.4)	−0.04
**Medication at discharge**						
β-Blocker	903 (87.0)	886 (85.6)	0.04	699 (86.0)	699 (86.0)	0.00
Statin	1018 (98.4)	982 (95.2)	0.19	785 (98.0)	767 (96.3)	0.04
RAAS blocker	962 (93.0)	887 (86.0)	0.23	739 (92.0)	710 (89.5)	0.07
Proton–pump inhibitor / H2–receptor blocker	833 (80.5)	758 (73.3)	0.17	620 (77.2)	621 (77.2)	0.00
P2Y_12_ inhibitor switching after discharge	79 (7.6)	23 (2.2)	0.24	22 (2.7)	18 (2.2)	0.05
Time–to–switch, days	45 (6, 131)	93 (12, 171)	0.13	98 (60, 136)	86 (61, 111)	0.05
Duration of DAPT, days	369 (36)	360 (60)	0.18	351 (77)	359 (63)	−0.03
Medication possession ratio (1 year), %	68 (31)	71 (29)	−0.10	76 (28)	77 (27)	−0.04

*Data summarized as mean (SD), median (IQR), and n (%), as appropriate.*

* *Diagnosis of cancer more than 3 years before the index ACS*.

## Discussion

Among patients with ACS in this contemporary multicenter registry, after adjusting for fixed- and time-varying confounders, ticagrelor was not associated with a significantly greater risk of major bleeding compared with clopidogrel. Importantly, notwithstanding the marginally increased risk of bleeding among elderly patients and those with previous bleeding receiving ticagrelor, exploratory analyses suggested that ticagrelor significantly reduced the risk of MACE, resulting in a favorable net clinical benefit compared with clopidogrel. Interestingly, these findings underline the inability of ITT analysis for dealing with selection bias resulting from differential non-adherence with P2Y_12_ inhibitors in daily practice. We hypothesized that our findings could explain the raised concerns from recent studies regarding the actual net clinical benefit of ticagrelor over clopidogrel.

Clinical trials, observational and basic research all have contributed critically relevant information to elucidate the efficacy and overall safety of ticagrelor in patients with ACS (3–6). In the PLATO trial, ticagrelor, compared with clopidogrel, was associated with similar total major bleeding according to the study definition but increased nonCABG and nonprocedure-related major bleeding (3). Similarly, the current findings are plausibly consistent with the more consistent, faster, and stronger inhibition of platelet aggregation achieved by ticagrelor in comparison to clopidogrel (3, 4). In this regard, despite ticagrelor-treated patients being generally younger with less comorbidity burden than clopidogrel users, the increased risk of bleeding with ticagrelor vs. clopidogrel, even after multivariable adjustment, may likely be explained, at least in part, by the relatively higher exposure to ticagrelor compared to clopidogrel in the current study. Against this background, recent evidence from observational studies (4–11, 28), and small randomized clinical trials (RCTs) (12, 13), have given rise to serious concerns about whether the favorable net clinical benefit of ticagrelor vs. clopidogrel in PLATO trial, actually holds in contemporary clinical practice. In the same direction, in a recent network meta-analysis of 12 randomized-controlled trials **(**RCTs), despite a significant mortality benefit, ticagrelor was associated with an increased risk of major bleeding compared with clopidogrel (29). In contrast, direct pairwise comparisons revealed that ticagrelor was not significantly associated with a greater risk of major bleeding vs. clopidogrel (hazard ratio, 1.38; 95% CI, 0.97–1.98) (29), which completely echoes our findings. Likewise, in a small clinical trial conducted in the East Asian population, while ITT analysis demonstrated a significantly higher incidence of major and fatal bleeding in the ticagrelor group than in the clopidogrel group; neither a significantly higher incidence of MACE nor an increased risk of major BARC bleeding was found when comparing the use of ticagrelor vs. clopidogrel after lag-censoring, modified ITT analysis, or per-protocol analysis (12).

Our results are of special interest for everyday practice, as they confirm a reassuring safety profile of ticagrelor in a broad ACS population which is free from trial-related restrictions. Despite a sub-analysis from the Swedish Web-System for Enhancement and Development of Evidence-Based Care in Heart Disease Evaluated According to Recommended Therapies (SWEDEHEART) registry, more bleeding occurred with ticagrelor than with clopidogrel, as in our study, when patients with a history of major bleeding, prior to hemorrhagic stroke, and those receiving dialyses were excluded from the analysis, no significant difference in the risk of bleeding was found between treatment groups (5). These findings should be put into perspective with those from recent studies, some of them indicating a reduction in MACE in favor of ticagrelor (5, 16, 30, 31), others finding neither difference in mortality nor in MACE (7–11, 28), with the overwhelming majority observing an increased risk of bleeding with ticagrelor vs. clopidogrel (5–11). Nonetheless, the underlying reasons for these contradictory results are still unclear.

First, it could be argued that the improved performance of the new-generation DES in reducing stent thrombosis, besides the increasingly widespread use of intra-coronary imaging techniques to optimize stent deployment, could have blunted the potential advantages of potent P2Y_12_ inhibitors over clopidogrel in reducing thrombotic events. On the other hand, despite PLATO, there was no signal of an age-by-treatment interaction for the risk of major bleeding (32), among elderly patients with non-ST elevation ACS in the POPular AGE trial, ticagrelor in comparison with clopidogrel, significantly increased bleeding complications without reducing MACE risk (13). Likewise, in a recent analysis from the SWEDEHEART, including elderly post-MI patients, ticagrelor was associated with a higher risk of bleeding and death compared with clopidogrel (6). Accordingly, it could be hypothesized that the mixed findings reported in those studies could stem from the heightened risk of bleeding associated with ticagrelor, compared with clopidogrel among the more co-morbid and frail older patients post-ACS encountered in everyday practice.

Against this background, our study suggests that previous conflicting findings may be explained, at least in part, by the methodological heterogeneity across studies. It should be noted that safety concerns surrounding ticagrelor use have primarily arisen from retrospective analyses of administrative claims data, mostly based on the ITT assumption (5–9). An accurate assessment of exposures and outcomes over time is not only a critical step of observational research but also a non-negligible source of bias, particularly in retrospective studies (21). In this context, although data linkage already offers a feasible approach to effectively capture real-world clinical outcomes from retrospectively collected data, there is certainly strong evidence for the limited accuracy of medical claims in identifying bleeding events, compared with that based on physician adjudication (33). On the one hand, if we assume that non-adherence to medication is typically ignored by ITT analysis, this approach may not reflect the real use of P2Y_12_ inhibitors in routine practice, which can result in biased estimates due to differential exposure misclassification (21). In real-world settings, non-adherence largely differs from that in RCTs. Of note, differential nonadherence with P2Y_12_ inhibitors is a frequently encountered issue in RCTs, but also in daily practice (13, 34). Interestingly, among 66,870 patients included in 4 recent RCTs, the risk of premature discontinuation was 25% higher for ticagrelor-treated patients compared with those receiving the comparator (clopidogrel/placebo) (34). In view of the existing evidence, differential nonadherence with P2Y_12_ inhibitors is a common threat in clinical practice, but as our study suggests, it should also be deemed as a potential source of selection bias to deal with in the observational studies based on the ITT principle (7–9). Nevertheless, despite the inherent limitations of ITT analysis in observational research, not enough attention has been paid to assessing the comparative safety and effectiveness of P2Y_12_ inhibitors according to the on-treatment principle. Furthermore, the time-varying nature of exposure has been systematically ignored by recent retrospective studies in the field, with the consequent risk of selection bias (9, 31).

### Strengths and Limitations

The implications of this study are 2-fold. While the observed results may help inform decision-making in clinical practice, our findings also come to fill some gaps and weaknesses in the evidence. Interestingly, this study suggests that in the presence of differential medication non-adherence, the “on-treatment” approach clearly outperforms the standard ITT analysis in the observational research, by minimizing the likelihood of selection bias. Likewise, we demonstrated that the ITT approach led to differential exposure misclassification, which skewed the relative risk of bleeding in favor of ticagrelor over clopidogrel in our sample. Therefore, following the ITT principle does not provide the most accurate estimate, as the level of nonadherence increases. However, the critical issue in conducting “on-treatment” analysis is the methodological challenge of adjusting for time-varying confounders, especially when censoring is highly unbalanced (24, 25). This approach is heavily reliant on data granularity and requires an accurate model specification that may not be routinely accessible using data linkage (22, 33). Furthermore, we use a whole arsenal of statistical tools to deal with various sources of biases, including selection bias, information bias, and both measured and unmeasured confounding bias. To date, although methods for addressing potential unmeasured confounding have been less developed in the clinical research (27), it is noteworthy that the findings of IV analysis in our sample were fully consistent with those from the primary analysis.

The current study has also several limitations that should be acknowledged. First, despite major efforts to control the different sources of biases, residual or unmeasured confounding could persist. Second, the study design precludes evaluating antithrombotic regimes other than standard DAPT, including aspirin-free or abbreviated DAPT strategies. Third, IPCW is a heavily data-reliant method, which is prone to substantial bias with a high level of missing data, small sample sizes, or large switching proportions. Nevertheless, none of these shortcomings apply to the present study. Finally, although, it could be argued that the observed reduction in MACE risk with ticagrelor vs. clopidogrel could stem from the widespread use of revascularization in the former group, a more in-depth analysis did not confirm that hypothesis. On the one hand, after formal blinded adjudication of outcomes, the hierarchical distribution of MACE events, according to the time of the first occurrence of any of the components of the composite endpoint, showed that MACE risk was mainly driven by the occurrence of death and MI, while only 13% of events were directly related to revascularization. On the other hand, PSM analysis showed that there was a consistent relative risk reduction for MACE with ticagrelor vs. clopidogrel when both treatment groups were balanced regarding the revascularization strategy. Nevertheless, results from the secondary outcomes should be deemed exploratory as this study was underpowered to definitely assess efficacy.

## Conclusion

Among patients with ACS in contemporary clinical practice, ticagrelor did not increase the overall risk of major bleeding but consistently proved superior to clopidogrel in reducing major ischemic events and mortality. Interestingly, this study demonstrates that the IPCW method may be particularly suited for assessing time-to-event outcomes in the presence of differential medication non-adherence, which otherwise would not be explored with the standard ITT approach. Nevertheless, further research is warranted to assess the comparative safety and effectiveness of P2Y_12_ inhibitors in high bleeding risk populations according to the “on-treatment” principle, while accounting for the time-varying nature of exposure in routine clinical practice.

## Data Availability Statement

The original contributions presented in the study are included in the article/Supplementary Material, further inquiries can be directed to the corresponding author.

## Ethics Statement

The studies involving human participants were reviewed and approved by the Regional Research Ethics Committee of Andalusia and the Institutional Review Boards at each participating center approved this study. The patients/participants provided their written informed consent to participate in this study.

## Author Contributions

MA-D, JG-R, and JA: had full access to all data in the study and took responsibility for the integrity of the data and the accuracy of the data analyses. MA-D, JG-R, and JA: study concept and design. JG-R, MA-D, JA, and JR: analysis and interpretation of data. JG-R and JA: blinded-endpoint adjudication and sites #1 and #2. JR and JS: sites #3 and #4. IF and JC: sites #5 and #6. MA-D: drafting of the manuscript. MA-D: obtained funding. JG-R, MA-D, and JA: study supervision. All authors had critical revision of the manuscript for important intellectual content. All authors had access to relevant data, acquisition of data and participated in the drafting, review, and approval of the final manuscript for submission.

## Funding

Andalusian Health Authorities sponsored the ARIAM-Andalucía registry with no funding from any agency in the public, commercial, or not-for-profit sectors. Data collection for this study was partially supported by an unrestricted research grant from AstraZeneca (ESR-17–13127), which had no role in the study design, data collection, data analysis, data interpretation, manuscript writing, or decision to submit.

## Conflict of Interest

In the past 3 years, MAD has received honoraria for lectures from Eli Lilly Co, Daiichi Sankyo, and AstraZeneca, and reported receiving consulting fees from AstraZeneca, and Daiichi Sankyo. The remaining authors declare that the research was conducted in the absence of any commercial or financial relationships that could be construed as a potential conflict of interest.

## Publisher's Note

All claims expressed in this article are solely those of the authors and do not necessarily represent those of their affiliated organizations, or those of the publisher, the editors and the reviewers. Any product that may be evaluated in this article, or claim that may be made by its manufacturer, is not guaranteed or endorsed by the publisher.
