# Supplementary material for: Comparative Safety and Effectiveness of Ticagrelor versus Clopidogrel in Patients With Acute Coronary Syndrome: An On-Treatment Analysis From a Multicenter Registry
Source: Front Cardiovasc Med. 2022 May 27;9:887748. doi: 10.3389/fcvm.2022.887748 (PMC9197128; doi:10.3389/fcvm.2022.887748)
Supplement: Supplementary file 1 [file Data_Sheet_1.pdf]

Supplementary Material

Page

1 Supplementary Methods 1. Inclusion and Exclusion Criteria 2

1.1 Inclusion criteria 2

1.2 Exclusion criteria 2

2 Supplementary Methods 2. Additional Information on Methods 3

2.1 Outcomes definitions 3

2.2 Data sources. Exposure and Outcome Ascertainment 4

2.3 Sample size estimation 6

2.4 IPCW Fully Adjusted Cox Proportional Hazards and Fine-Gray Regression Models 7

2.5 Sensitivity analysis 13

2.5.1 Subgroup analysis of major bleeding 13

2.5.2 Landmark analysis of major bleeding 13

2.5.3 Propensity Score Matching Analysis 14

2.5.4 Instrumental Variable Analysis 14

2.5.5 Intention-to-treat Analysis 15

3 Supplementary Tables and Figures 17

3.1 Supplementary Tables 17

Table S1. Duration of DAPT according to the $P2Y_{12}$ inhibitor-adherence pattern 17

Table S2. Timing of permanent DAPT discontinuation 18

Table S3. Reasons for DAPT discontinuation 19

Table S4. Results of the Propensity Score Matching Analysis 20

Table S5. Results of Instrumental Variable Analysis 21

Table S6. Baseline data for patients discharged on clopidogrel or ticagrelor by calendar year 22

Table S7. Primary and secondary outcomes according to calendar day 23

Table S8. Intention-to-treat analysis of 1-year outcomes in the overall study cohort and the propensity-score matched patients 24

3.2 Supplementary Figures 26

Figure S1 Temporal trends of ticagrelor and clopidogrel use during the study period in the entire cohort and stratified by participating centers 26

Figure S2. Subgroup analysis of BARC type 3 or 5 bleeding 26

Figure S3 Landmark analysis of major bleeding events at 30 days 28

Figure S4 Standardized mean difference plot for covariates before and after matching 29

1 Supplementary Methods 1: Inclusion and Exclusion Criteria

1.1 Inclusion criteria (All of the following):

- Age ≥ 18 years.
- Patients discharged alive after the index acute coronary syndrome (ACS) hospitalization.
- Patients discharged on dual antiplatelet therapy (DAPT) for at least 12 months, combining low-dose aspirin (75–100 mg/day) and a $P2Y_{12}$ receptor inhibitor, either clopidogrel or ticagrelor.

1.2 Exclusion criteria (Any of the following):

- Age < 18 years.
- Subjects who died during the index admission.
- Hypersensitivity or contraindication to ticagrelor, clopidogrel or aspirin.
- Patient discharged without a $P2Y_{12}$ inhibitor or treated with prasugrel.
- Need for chronic oral anticoagulation therapy, or use of strong cytochrome P-450 3A inhibitors or inducers, either during the index admission or after hospital discharge.
- History of major bleeding within 6 months prior to the index admission.
- History of previous intracranial haemorrhage (ICH) at any time.
- Cancer diagnosis within the 3 years preceding the index admission.
- Known severe liver disease.
- Failure to obtain informed consent from participants.
- Any condition or diagnosis which in the opinion of the investigator would make patients less likely to be prescribed a more potent antiplatelet agent (e.g., frailty, dementia…).
- Patients lost-to follow-up, or with missing data for other reasons.
- Diagnosis other than ACS at hospital discharge (i.e., acute myocarditis, Takotsubo syndrome, pulmonary embolism, acute aortic syndrome…).
- Index myocardial infarction (MI) secondary to an ischaemic imbalance (namely type 2 MI according to the third universal definition) in instances of myocardial injury with necrosis, where a condition other than coronary artery disease contributes to an imbalance between myocardial oxygen supply and/or demand (e.g., anaemia, sepsis, tachyarrhythmia, bradyarrhythmia, hypotension, heart failure…).

2 Supplementary Methods 2: Additional Information on Methods

2.1 Outcomes definitions

Primary outcome

Major bleeding. First occurrence of Bleeding Academic Research Consortium (BARC) type 3 or type 5 bleeding, up to 1 year after the index admission.

BARC category	Definition
**Type 3**	
**3a**	- Overt bleeding plus Hgb drop of 3 to < 5 g/dL* (provided Hgb drop is related to bleed).<br>- Any transfusion with overt bleeding
**3b**	- Overt bleeding plus Hgb drop ≥ 5 g/dL* (provided Hgb drop is related to bleed).<br>- Cardiac tamponade.<br>- Bleeding requiring surgical intervention for control (excluding dental/nasal/skin/haemorrhoid).<br>- Bleeding requiring intravenous vasoactive agents.
**3c**	- Intracranial haemorrhage (does not include microbleeds or haemorrhagic transformation; does not include intraspinal), subcategories confirmed by autopsy, imaging, or lumbar puncture.<br>- Intra-ocular bleed compromising vision.
**Type 5**	
**5a**	- Probable fatal bleeding (clinically suspicious, but not directly observed as the cause of death).
**5b**	- Definite fatal bleeding (directly observed by either clinical specimen [blood, emesis, stool, etc.] or imaging, or confirmed on autopsy).

Note: Platelet transfusions should be recorded and reported but are not included in these definitions until further information is obtained about the relationship to bleeding outcomes.
* Corrected for transfusion (1 unit PRBC or 1 unit whole blood ≈1 g/dL Hgb).
Abbreviation: Hgb, haemoglobin.

Secondary outcomes

Major Adverse Cardiac Event (MACE): Defined as the composite of all-cause mortality, non-fatal MI, non-fatal stroke, or urgent target lesion revascularization within1-year after the index ACS.

- **All-cause mortality**
  - **Vascular death**, defined as cardiovascular (CV) and cerebrovascular death. These include death due to MI, sudden cardiac (including unwitnessed) death, heart failure, stroke, bleeding of cardiovascular origin, or other vascular causes (peripheral artery disease, aortic syndromes…).
  - **Non-cardiovascular death**, defined as those deaths with a specific cause that is not thought to be CV in nature.
  - **Unknown death**, defined as a death not attributable to any other category because of the absence of any relevant source documents following a complete and thorough clinical investigation. Such deaths are classified as CV for endpoint determination.

- **Myocardial infarction:** defined as the occurrence of a spontaneous MI due to a primary coronary event related to atherosclerotic plaque rupture, ulceration, fissuring, erosion, or dissection with resulting intraluminal thrombus in one or more of the coronary arteries, leading to decreased myocardial blood flow or distal platelet emboli with ensuing myocyte necrosis (type 1), or MI caused by either PCI (type 4a), stent restenosis (type 4c), or cardiac surgery (type 5), according to the Third Universal definition of MI.

- **Stroke:** symptoms, pathological or neuroimaging evidence of acute newly onset focal neurologic deficit last for more than 24 hours, resulting from a presumed sudden abnormality of the blood supply after excluding other non-ischemic reasons.

- **Urgent target lesion revascularization (uTLR)**: any non-elective repeated percutaneous or surgical revascularization of the target lesion performed for restenosis or other complication up to 1 year after the index ACS.

Definite/probable stent thrombosis (ST): defined according to the Academic Research Consortium (ARC) classification. Stent thrombosis was classified as early (occurring in the first month after PCI) or late (occurring between 1 and 12 months after PCI). In addition, early ST was subdivided into acute (within first 24hours) and subacute (between 1 and 30 days).

Net Adverse Clinical Event (NACE): defined as the composite of safety (BARC types 3 or 5 bleeding) and efficacy (MACE) outcomes within 1 year following index ACS.

2.2 Data sources, Exposure and Outcome Ascertainment

Data Sources

The **Andalusian Public Healthcare System (APHS)**, as part of the Spanish National Health System (NHS), is funded from taxation to provide comprehensive health care coverage to all individuals of Andalusia (Southern Region in Spain). All citizens in Andalusia have a unique personal identification number, which is linked to their electronic medical records (EMR). The EMRs for each citizen in the APHS are stored in a centralized computer system, the **Andalusian Public electronic Healthcare System (AeHS)**, called **DIRAYA** (arabic for knowledge), which integrates all the health data from Primary Care System, Emergency Services, Mental Health Services, Hospitalizations, and the outpatient Specialized Care.

Outcome Assessment

In both the ARIAM-Andalucía and CREA registries, over 150 variables on demographics, comorbidities, angiographic and procedural characteristics, medications, and clinical events, are prospectively captured through a standardized electronic case report form, which is filled out on a web-based database platform. After discharge data were captured during the outpatient visits scheduled at 30±10 days and 365±30 days after the index admission date, and by semi-structured telephone interviews with patients or relatives. The phone interviews consisting of a standardized questionnaire asking for side effects and self-reported adherence to prescribed medications, hospital readmissions, and bleedings. Previous being put forward for formal adjudication based on prespecified criteria, patient self-reported information underwent an

extensive validation process. This involved an extensive and detailed review of corresponding EMRs, which included hospital files, and, if necessary, general practitioner, outpatient clinic, or pharmacy records.

All hospital admission identified from the patients´ EMRs, using their APHS number as a unique personal identifier, were screened for potential identification of clinical outcomes. For this end, we did not use the internationally accepted codes to extract all diagnoses (ICD-10) and procedures (OPCS-4) generated or performed during each hospitalization episode. By contrast, any relevant information that could enhance accuracy of endpoint ascertainment, including laboratory, imaging, and procedural data, was flagged in the EMRs by manual inspection of each hospitalization or outpatient episode during the study period.

The suspected outcomes ultimately underwent formal blinded adjudication based on prespecified criteria. Event adjudication was performed by consensus of two experienced investigators at each center, that were blinded to the calendar year and $P2Y_{12}$ inhibitor exposure, using original anonymized adjudication pack documents. Discrepancies were resolved through a consensus discussion with a third consultant cardiologist not involved in the study. The study principal investigator´s hospital served as the central coordination center for event adjudication. Random IDs generation ensured blinded sequence. If deemed necessary vital status and specific causes of death were obtained from the Spanish Civil Registry and Death Certificates Records, respectively. Following formal adjudication of clinical outcomes and exposure classification, data were definitely incorporated into a web-based database platform with password protected files for further data analysis.

Exposure Ascertainment

The **AeHS** includes a centralized **Electronic Drug Prescription and Dispensation Registry** (**EDPR**), called **Receta XXI** that connects doctor's offices and pharmacies in real time. This system serves as a real time prescription monitoring tool, which provides, previous consent, instant access to the patient's medication history. For the present analysis, exposure was defined by a prescription fill in the EDPR for clopidogrel or ticagrelor within first 7 days after admittance. Adherence was calculated based on prescription fill dates and days' supply. To this end, two measures of exposure were calculated assuming an intended 12-month DAPT course: **adherence**, and **switch**.

First, to account for **medication adherence** during the follow-up, we estimated time-dependent cumulative drug exposures from hospital admission until time of death, drug discontinuation, drug switching, or censoring, establishing the $P2Y_{12}$ inhibitor treatment status at different time points according to the on-treatment principle (i.e., the $P2Y_{12}$ inhibitor actually received). Adherence was estimated using the **medication possession ratio** **(MPR)** metric, defined as the proportion of a time period where a medication supply is available divided by the total number of days in the observation time period, expressed as a percentage, according to the formula bellow:

$$\textbf{MPR}= \left(\frac{\text{Sum of days´ supply for all fills in period}}{\text{Total number of days in period}}\right) \text{ x } 100\ (\%)$$

DAPT cessation was defined as withdrawal of $P2Y_{12}$ inhibitor therapy, assuming full adherence to aspirin. Different patterns of non-adherence to DAPT regimens were considered: permanent discontinuation, interruption, and disruption. **Permanent DAPT discontinuation** was defined as permanent drug withdrawal if DAPT was never resumed after discontinuation, while **DAPT interruption** was defined as brief (1-5 days), or temporary (5-14 days) cessation of medication that was reinstituted within 14 days. **DAPT disruption** was a defined as a form of permanent discontinuation of DAPT due to bleeding or non-adherence. DAPT non-adherence was defined as cessation of antiplatelet medication without any side effect, clinical event, or physician-based recommendation (**Tables S1-S3**).

Second, **$P2Y_{12}$ inhibitor switch** was defined as ≥ 1 outpatient prescription fills for a different $P2Y_{12}$ inhibitor agent from the first fill, within 365±30 days after the index event (**Table S4**).

2.3 Sample size estimation

The primary hypothesis of the study was that the safety profile of ticagrelor is not unacceptably worse compared to that of clopidogrel in terms of major bleeding risk. Based on previous studies including similar populations, an expected 1-year rate of major bleeding (BARC type 3 or 5 bleeding) of 5.6% was assumed for both groups. Therefore, assuming a type I error of 5% with a two-sided 95% confidence interval (CI)—equivalent to a one-sided 97.5% CI with 2.5% level of significance—a sample size of 1776 subjects (888 patients per group, and a total of 85 major bleeding events) would provide at least 80% power to detect the upper bound limit (UBL) of the two-sided 95% CI for the point estimate in terms of relative risk (i.e., the adjusted subdistribution hazard ratio, aSHR) of BARC major bleeding for ticagrelor *vs.* clopidogrel not to exceed 2.10. This margin was selected on the basis of the existing literature at the time of study design and represents the maximum acceptable or permissible excess risk of major bleeding that would be expected for ticagrelor relative to clopidogrel in a real-world setting. Therefore, if the UBL was < 2.10, then the null hypothesis that clopidogrel is safer than ticagrelor with regards to bleeding risk would be rejected. We included additional size adjustments to minimize unequal distribution of participants among groups due to attrition bias and differential switching rates during follow-up. Thus, estimating overall rates of 9% for switching and 13% follow-up loss, this led to a final sample size of **1900 subjects** (950 patients in each group, with 104 major bleeds expected) to be sensible with at least 85% of power to detect the UBL of the two-sided 95% CI of the aSHR for major bleeding below 2.10.

Upper bound 95% confidence interval limit selection and interpretation

The upper bound 95% CI limit comes to be the difference that would be clinically acceptable to conclude that there is no difference between treatments with respect to the event of interest (i.e., to demonstrate safety of ticagrelor compared to clopidogrel with regards to the risk of major bleeding). The UBL was selected based on data from previous real-world studies with antiplatelet agents in ACS. In general, relative scales, such as the hazard ratio, are usually preferred over absolute scales, such as the arithmetic absolute risk difference, to fix the margin in case of unanticipated dissimilarities in observed and expected event rates. Under the above-mentioned considerations, the study would be statistically sensible enough to test safety if the UBL of the two-sided 95% CI for the aSHR of major bleeding for ticagrelor versus clopidogrel was below the established margin of 2.10, indicating that ticagrelor is "at least as safe"—but

not inferior—as clopidogrel in terms of major bleeding risk. Statistical test for power analysis assumed unweighted *log-rank* test for estimation, uniform accrual, and different rates of: drug discontinuation and switching, loss to follow-up (drop-outs) and allocation ratios, leading to unequal group sizes.

In order to adjust for ascertainment bias, one could anticipate an additional reduction in the sample size when conducting sensitivity analysis using propensity score methods. Therefore, the potential impact of this approach on statistical power was, as far as possible neutralized by using a step-by-step adjustment procedure for the matching process. This method aims to balance loss of power versus increase bias, thus minimizing a size-limited paired sample which could lead to study results being underpowered to test the primary hypothesis.

2.4 IPCW Fully Adjusted Cox Proportional Hazards and Fine-Gray Regression Models

The Inverse probability of censoring weighting (IPCW) method

In the present analysis, an IPCW approach was implemented to address informative censoring introduced by a differential nonadherence with $P2Y_{12}$ inhibitor therapy (different rates of drug discontinuation and switching between the study groups). This method provides an unbiased estimator of censoring distribution conditional on covariates when censoring is not random, but rather depends on covariates and time. The key components in the IPCW approach are the estimated weights that later will be introduced into the survival models to diminish informative bias due to differential medication nonadherence. Furthermore, the survival models included robust variance estimators to account for the additional variability introduced by estimating the IPWs.

Importantly, to provide reliable and accurate estimates when applying the IPCW approach, we should take special care to satisfy four key assumptions. First, IPCW is reliant upon the assumption that all the data are available and appropriately measured on all baseline (time-fixed) and potential time-dependent predictors that could influence the probability of drug discontinuation or switching, and the occurrence of the event of interest. This assumption is commonly referred to as the "no unmeasured confounders" (NUC) assumption. In the present study, this assumption was met since according to the study protocol, the eCRF (electronic case report form) was explicitly designed to capture all covariates that could be associated with drug discontinuation and switching. These included both "static" predictors collected at baseline, and other potential time-dependent covariates that could be captured during the follow-up. Such detailed information was available throughout the duration of the study because the CREA registry was designed as an investigator-initiated branch of ARIAM-Andalucía registry, aimed at providing relevant information on incidence, timing, determinants and clinical significance of switching between $P2Y_{12}$ inhibitors in post-ACS patients managed according to contemporary recommendations. Second, the other major issue to be considered when applying the IPCW method is that this type of analysis may introduce additional bias when switching occurs frequently, or in the case of small samples and low incidence of events in the subjects who switch treatments. Nevertheless, neither the rate of switching nor the sample size represented a drawback to deal with in the present study. Third, to satisfy the assumption of NUC, weights should be calculated from a correctly specified model that includes all baseline and time-varying covariates predictive of both treatment nonadherence and outcomes, which

requires extensive data collection, as the current study did. Finally, the probability of treatment switching must always be less than one for all possible predictors combinations, otherwise weights cannot be estimated.

Estimation of weights

To obtain the censoring weights according to IPCW method, the likelihood of remaining uncensored (in the case at hand, do not change the initially assigned $P2Y_{12}$ inhibitor at hospital discharge, or do not discontinue DAPT before 12 months from index admission) was estimated by means of two separate Cox regression models. The first model used only baseline (time-invariant predictors) covariates, while the second one was fitted for both relevant baseline and time-dependent covariates, to estimate the probability of changing the exposure status over time. Importantly, probabilities should be generated separately for each treatment group, since in the current study drug switching was expected to be not random, but rather likely to be attributable to factors that could vary depending upon the exposure status, the comorbidity burden, or the occurrence of clinical outcomes of interest. Afterwards, the stabilised weights for each person-time interval are derived by the ratio of the estimated probability that each patient remained uncensored (i.e., stay on the initial $P2Y_{12}$ inhibitor), conditional on time and past treatment, divided by the probability that the patient stay on the initial $P2Y_{12}$ inhibitor given time, past treatment, and both baseline and time-dependent predictors. Subsequently, patients who switched treatments will have lower weights (as IPCW method rely on weights according to the "inverse" probability of being censored) that patients who did not. On the other hand, patients who stay on the initially assigned treatment (uncensored) will have a weight equal to 1. The list of baseline and time-dependent covariates used in the calculation of the stabilized weights to obtain robust IPCW estimators for clopidogrel and ticagrelor exposures are depicted below:

- **Clopidogrel exposure predictors:**

  Baseline covariates: calendar year, age (continuous variable), sex, history of prior stroke, chronic kidney disease, previous bleeding, previous MI, history of anaemia, ACS type, CRUSADE score (continuous variable), GRACE score (continuous variable), reperfusion strategy, and multivessel disease.

  Time-dependent covariates: MPR (continuous variable), and the occurrence of thrombotic events (MI, ST, or unplanned TLR) during follow-up.

- **Ticagrelor exposure predictors:**

  Baseline covariates: calendar year, age (continuous variable), sex, history of prior stroke, chronic kidney disease, previous bleeding, previous myocardial infarction, history of anaemia, ACS type, CRUSADE score (continuous variable), GRACE score (continuous variable), reperfusion strategy, and multivessel disease.

  Time-dependent covariates: MPR (continuous variable), and occurrence or dyspnea or bleeding during follow-up.

Time-varying exposure

Compliance is frequently related to study outcomes independently of the exposure itself. However, this would lead to problems with data interpretation if noncompliance would be related to specific side effects of the medication under evaluation, thus leading to a differential nonadherence between groups. Consequently, this drawback may give rise to a false or misleading impression that the medication which is discontinued would seem to be better than it actually would be in daily clinical practice. In order to account for issues generated when noncompliance occurs in a no-random fashion (differential nonadherence), we performed a **lag-censoring analysis** while adjusting for drug switching. This method aiming at dismissing the dilution effect on intention-to-treat (ITT) estimates that results when drug discontinuation is unevenly distributed among the study groups.

In short, with lag-censoring analysis the follow-up is censored at some time—the lag-time or lag-period—after treatment was discontinued. Consequently, if either the time to drug cessation (which is, the feature that precludes the observation of the event of interest), or drug discontinuation itself is related to the event under investigation, this would imply that non-compliant patients, whose follow-up is censored, could have an incidence rate of the event which could differ from that of the patients who remain under observation (compliant subjects). If such a situation occurs, censoring at "some" time (lag-period) after discontinuing treatment may generate bias. However, the key issue in keeping with the lag-censoring analysis rely on the **assumption of independent censoring**. This assumption implies that both time-to-censoring and time-to-event are independent, which implies that survival times for censored and uncensored individuals is comparable, and removal of censored subjects from analysis would yield an unbiased estimate of time-to-event. Besides the independent censoring assumption, another relevant feature to account for in conducting lag-censoring analysis is the **choice of the lag time**, to cope with the temporal relationship between DAPT cessation and the occurrence of future ischemic events. The lag time may be selected depending on both the pharmacokinetic properties of $P2Y_{12}$ inhibitors (onset/off-set effect), and the temporal relationship between DAPT discontinuation and the occurrence of potential thrombotic events (rebound effect). In this study, the lag time was selected based on the available evidence showing a 2-fold increase in the risk of death or MI in the first 90 days following clopidogrel withdrawal in post-MI patients.

Weighted Cox proportional hazards and Fine-Gray regression models

The stabilised weights were incorporated into the Cox proportional hazards or the Fine-Gray regression models, as appropriate, in order to estimate the measures of association between exposure and outcomes over time, accounting for informative censoring due to non-adherence (premature discontinuation or switching) with $P2Y_{12}$ inhibitor therapy. In addition, hospitals were entered as a random cluster-effect to account for both the potential variability in clinical performance across centres, and that introduced by the stabilised weights itself. Finally, the survival models were fitted for each clinical outcome accounting for confounding by selection of significant predictors in the univariate analysis that showed association with the outcome of interest, and those identified as potentially relevant based either on clinical experience, or the published literature. Prior to be included into the models, the best fitting functional form of continuous predictors was explored using multivariable fractional polynomials. Thereafter, no

transformation was required as the conformity with linear gradient was met for all continuous covariates.

The modelling process comprised a stepwise approach with a threshold set at $P < 0.10$ for entering and $P > 0.05$ for removing variables from the model. The final regression model was selected by minimizing the Akaike information criterion (lower values indicate a better fit), accounting for multicollinearity to achieve the most parsimonious model while retaining the highest level of explanatory ability. To this end, the individual components of CRUSADE and GRACE risk scores affected by serious multicollinearity were removed from the models, unless this resulted in a sizeable decrease in the predictive performance. Therefore, whenever possible, both CRUSADE and GRACE scores were included into the survival models as continuous predictors to avoid potential overfitting that may be expected had each of their individual components have been added separately. Model assumption was evaluated using Schoenfeld residuals. Discrimination and calibration of the resulting models was assessed by the c-statistic and calibration slope, respectively. Multivariate models for each outcome were adjusted for baseline (time-independent) covariates as follows:

- **Primary Outcome**
  - **BARC major bleeding:** covariates in model, n= 10; observed events, N= 106; ratio (n/N) 1:10.6 (rule of thumb). Covariates: $P2Y_{12}$ inhibitor, body mass index (continuous), history of previous bleeding or anaemia, thrombolytic therapy, vascular access, use of GP IIb/IIIa inhibitors, MPR (continuous), CRUSADE score (continuous), and use of proton pump inhibitors (PPI).

    Model performance: c-statistic 0.80; 95% CI (0.75–0.83)

    calibration slope 1.05; 95% CI (1.04–1.06)
- **Secondary Outcomes**
  - **MACE:** covariates included, n= 14; observed events, N= 198; ratio (n/N) 1:14. Covariates: $P2Y_{12}$ inhibitor, sex, age (continuous), previous diagnosis of cancer (> 3 years before index admission), GRACE and CRUSADE risk scores (both as continuous predictors without transformation—per 10-point increase—), ACS type, multivessel disease and completeness of revascularization, left ventricular ejection fraction (LVEF) at discharge (continuous), duration of DAPT (continuous covariate), and MPR (continuous covariate).

    Model performance: c-statistic 0.76; 95% CI (0.72–0.81)

    calibration slope 1.10; 95% CI (1.13–1.16)
  - **Individual components of MACE.** Owing to the relatively small number of events observed for some individual components of MACE, to prevent overfitting as much as possible, models were adjusted to cope with the general rule of thumb that there should be at least 10 events for each predictor variable (Event Per Variable, EPV) entered the regression models (one in ten rule). This rule was relaxed from 10 to 8 EPV in the case of non-fatal stroke, after excluding intracranial haemorrhages and secondary haemorrhagic transformation of ischaemic stroke. Therefore, the number of EPV in models evaluating each component of MACE was as follows: all-cause death (11 covariates; 105 deaths; EPV 10), non-fatal MI (7 covariates; 82 events; EPV 12), non-fatal stroke (3 covariates; 23 events; EPV 8); and urgent TLR (7 covariates; 66 events; EPV 10). Model performance:

All-cause death: c-statistic 0.85; 95% CI (0.82–0.88)
calibration slope 1.02; 95% CI (1.01–1.03)

Non-fatal MI: c-statistic 0.77; 95% CI (0.71–0.82)
calibration slope 1.18; 95% CI 1.10–1.22

Non-fatal Stroke: c-statistic 0.63; 95% CI (0.55–0.71);
calibration slope 1.20; 95% CI (1.11–1.30)

Urgent TLR: c-statistic 0.65; 95% CI (0.59–0.70);
calibration slope 0.94; 95% CI (0.68–1.40)

- **Definite/Probable ST**: (6 covariates; 59 events; ratio 1:10)
  c-statistic 0.68; 95% CI (0.60–0.75);
  calibration slope 0.97; 95% CI (0.64–1.40)
- **Vascular Death**: (6 covariates; 55 events; ratio 1:9)
  c-statistic 0.72; 95% CI (0.67–0.76);
  calibration slope 1.09; 95% CI (1.07–1.11)
- **NACE**: (22 covariates; 274 events; ratio 1:13). The final model was adjusted for $P2Y_{12}$ inhibitors, sex, history of diabetes, MI, stroke, peripheral artery disease, chronic kidney disease, cancer and major bleeding, GRACE and CRUSADE scores (as continuous predictors without transformations—per 10-point increase—), presence of multivessel disease, management strategy (PCI/CABG/medical treatment), vascular access approach, LVEF at discharge (continuous), duration of DAPT (continuous), and MPR (continuous). The pharmacologic background was also considered by accounting for use of GP IIb/IIIa inhibitors during PCI, and prescription of PPI, beta-blockers, RAAS blockers and statins at hospital discharge.

  Model performance: c-statistic 0.72; 95% CI (0.70–0.76)
  calibration slope 1.16; 95% CI (1.14–1.18)

A detailed list of covariates included into the fitted Cox proportional hazards and Fine-Gray competing risks regression models for the primary and secondary outcomes is depicted in the table below:

List of covariates included into the fitted models for the primary and secondary outcomes

Covariates	Primary Outcome	Secondary Outcomes							
	BARC major bleeding	MACE	All-cause death	Myocardial infarction	Stroke	uTLR	Definite/probable Stent Thrombosis	Vascular death	NACE
$P2Y_{12}$ inhibitor	✔	✔	✔	✔	✔	✔	✔	✔	✔
Age [a]	collinearity	✔	collinearity	collinearity	collinearity	collinearity	collinearity	collinearity	collinearity
Sex	collinearity	✔	✔	collinearity	collinearity	collinearity	collinearity	collinearity	✔
BMI [a]	✔	collinearity	collinearity	collinearity	collinearity	collinearity			collinearity
History of DM	collinearity	collinearity	collinearity	collinearity	collinearity	✔	✔	✔	✔
Previous of MI		collinearity	collinearity	collinearity	collinearity	✔		✔	✔
Previous Stroke	collinearity	collinearity	collinearity	collinearity	✔				✔
History of PAD	collinearity	collinearity	✔	collinearity	collinearity			collinearity	✔
History of CKD	collinearity	collinearity			collinearity	collinearity	collinearity	✔	✔
History of Bleeding	✔		collinearity					collinearity	✔
History of Anaemia	✔								
Cancer		✔	✔						✔
ACS type		✔	✔	✔	collinearity	collinearity	collinearity		collinearity
LVEF [a]	collinearity	✔	✔	collinearity		collinearity		collinearity	✔
MVD		✔	✔	✔		✔	✔	✔	✔
Revascularization		✔	✔	✔		✔	✔		✔
Radial	✔								✔
CRUSADE [a]	✔	✔	✔	✔	✔				✔
GRACE [a]		✔	✔	✔	collinearity	✔	✔	✔	✔
Thrombolysis	✔								
GP IIb-IIIa blockers	✔								✔
PPI	✔								✔
Beta-blockers		✔		collinearity				collinearity	✔
RAAS inhibitors		collinearity							✔
Statins		✔		collinearity				collinearity	✔
DAPT duration [a]		✔	✔	✔		✔			✔
MPR (adherence) [a]	✔	✔					✔	✔	✔
IPCW [b]	✔	✔	✔	✔	✔	✔	✔	✔	✔
Hospital-cluster [b] (random-effects)	✔	✔	✔	✔	✔	✔	✔	✔	✔

List of covariates included into the fitted models for the primary and secondary outcomes (continued)

Checklist stickers in light green indicate the covariates included in the final model for each outcome

During the modeling process multicollinearity become apparent due to statistics redundancy. Data redundancy selectively affected to single prognostic predictors whose information was already collected as integer values for the individual components of CRUSADE and GRACE integer-based risk scores. To this end, individual covariates suffering from multicollinearity were removed, since that information was already included into the models as a continuous predictor, by means of an integer-based risk score.

[a] Variables entered the models as continuous predictors, after fulfilling the linearity assumption by means of the multivariate fractional polynomials approach.

[b] Covariates providing doubly robust variance estimators.

Abbreviations: ACS, acute coronary syndrome; BARC, Bleeding Academy Research Consortium; BMI, body mass index; CKD, chronic kidney disease; CRUSADE, Can Rapid risk stratification of Unstable angina patients Suppress ADverse outcomes with Early implementation of the ACC/AHA guidelines; DM, diabetes mellitus; GP, glycoprotein; GRACE, Global Registry of Acute Coronary Events; DAPT, dual antiplatelet therapy; IPCW, inverse probability of censoring weighting; LVEF, left ventricular ejection fraction; MACE, major adverse cardiac event; MI, myocardial infarction; MPR, medication possession ratio; MVD, multivessel disease; NACE, net adverse clinical event; PAD, peripheral artery disease; PPI, proton pump inhibitors; RAAS, renin angiotensin aldosterone system ; uTLR, urgent target lesion revascularization;

2.5 Sensitivity analysis

A set of sensitivity analysis were performed to access robustness of the primary analysis.

2.5.1 Subgroup analysis of major bleeding

We performed exploratory analysis for the primary outcome across pre-specified subgroups according to (1) age ($\geq$80, or <80 years); (2) sex; (3) history of previous bleeding; (4) stroke; (5) anaemia; (6) CRUSADE score (categories $\geq$50, or <50 points); (7) creatinine clearance levels (categories $\geq$30, or <30 ml/min/1.73 m^2); (8) fibrinolytic therapy for STEMI patients; and (9) use of gastroprotective drugs at discharge. Bleeding outcome differences according to $P2Y_{12}$ inhibitor in each subgroup were performed with a formal test (Wald) for interaction of that subgroup with treatment in a Fine-Gray competing risks regression model which included treatment (exposure), the subgroup of interest, and the interaction term of both covariates (in a multiplicative scale). (**Figure S2**)

2.5.2 Landmark analysis of major bleeding

Landmark analysis of the primary outcome was performed to account for procedural-related bleedings during hospitalization and poor adherence with $P2Y_{12}$ inhibitors within first 30 days from index admission. Therefore, we examined the risk of major bleeding according to exposure in two separate periods: during the more acute (0–30 days after randomization) vs. the later period (30–360 days) of the study. For the first period, only bleeds that occurred within the first 30 days were included. For the latter period, all patients who survived to 30 days were included in the analysis, regardless of whether they had suffered a bleeding in the earlier period. The association between treatment and major bleeding was estimated both unadjusted and adjusting for confounding. (**Figure S3**). The risk of BARC type 3 or 5 bleeding was higher during the acute period, to subsequently decrease during follow-up. Neither incidence of major bleeding in the acute, nor in the later period was found to be different for ticagrelor- and clopidogrel-treated patients.

2.5.3 Propensity Score Matching Analysis

Propensity scores estimation

Third, to control for baseline differences between groups, ticagrelor and clopidogrel were compared in a propensity score–matched cohort. For this end, a non-parsimonious logistic regression model was used to estimate the probability of being discharged on ticagrelor versus clopidogrel based on baseline, angiographic and procedural characteristics. The potential confounding factors selected for estimating propensity scores (PS) were prespecified as follows: <u>Demographics and baseline comorbidities</u>: age, sex, body mass index (BMI), cardiovascular risk factors (smoking, hypertension, hyperlipidemia, and diabetes mellitus), history of stroke/TIA, MI, peripheral artery disease, heart failure (NYHA class>III), chronic kidney disease, chronic obstructive pulmonary disease, atrial fibrillation, history of previous major bleeding or anemia, previous diagnosis of cancer, and previous PCI or CABG surgery procedure. <u>Clinical presentation and procedural features</u>: index ACS diagnosis, CRUSADE and GRACE risk scores, creatinine clearance levels at admission (using Cockcroft-Gault equation), hemodynamic instability (Killip class ≥2), management strategy during index admission (invasive, medical treatment), vascular access approach, multivessel disease, revascularization at the index admission (PCI or CABG surgery), DES implantation, complete revascularization (in patients with multivessel disease), use of GP IIb/IIIa inhibitor and thrombolytic therapy for STEMI. <u>Medication at discharge</u>: proton-pump inhibitors (PPIs), statins, renin-angiotensin-aldosterone system antagonists, and beta-blockers.

The generated PS were screened for identification of extreme values, that could have led to large variance of the estimates, nevertheless no extreme tails were found, and trimming was not required. Then, patients on ticagrelor were matched with those who received clopidogrel using 1:1 nearest-neighbor matching selection without replacement using a fine-tuned selection of calipers. The caliper was gradually narrowed, starting from 0.2 to obtain satisfactory matching as indicated by an absolute standardized difference in means (SMD) less than or equal to 0.10 for all variables. Thus, the final caliper was chosen by trial-error to obtain a resulting matched sample size at least 75% of the original cohort, after weighting the potential loss of power (resulting from a severely reduced matched population) *vs.* increase bias (secondary to incomplete matching). Finally, a PS-matched sample of 801 well-balanced pairs with ticagrelor and clopidogrel were obtained, which represented almost 80% of the entire study cohort. (**Table S4,** and **Figure S4**).

2.5.4 Instrumental Variable Analysis

Unmeasured confounding is an important source of bias when estimating the effect of an intervention, treatment, or exposure in observational studies. In this context, instrumental variable (IV) methods surge to address treatment effects in settings where substantial unmeasured confounding is expected. Aimed at accounting for changes in clinical practice during the study period to reduce bias due to unmeasured confounders, we performed IV analysis in the overall cohort using the calendar year of index admission as a $P2Y_{12}$ inhibitor treatment-preference instrument. The IV may act like a natural randomization if it is highly correlated with treatment under investigation, but neither related to patient characteristics or other confounding factors, nor directly affecting the outcome of interest. Unlike PSM, IV analysis can adjust for both measured and unmeasured confounding effects at the same time, and hence, allows for the possibility of making causal inferences with observational data. Importantly, there are three assumptions for a variable to be chosen as a valid IV: (1) the

instrument must be associated with the exposure (treatment); (2) the instrument does not affect the outcome except through treatment; and (3) the instrument does not share any cause with the outcome (independent from the event of interest).

Previous studies implemented logistic regression modelling when using IV, as a proxy for time to event data with a binary outcome. However, this approach inevitably leads to survivorship bias because it does not consider follow up time and censoring. To perform IV modelling within a survival analytical framework, we may estimate the regression coefficients that might have been obtained from a Cox model, using a standard Poisson regression by splitting the data finely and specifying the model as having one rate parameter per time interval (piecewise). This way, the Cox model could therefore be formulated as a model with piecewise-constant baseline hazards, which is modelled by a timescale parameter for each time recorded (with "pieces" defined at each time point). This is an exchangeable model for the baseline rate parameters, thus using neither the ordering nor the absolute scaling of the times. Therefore, in practice, implementation of this approach requires that follow-up for each individual is split in `small pieces´ of follow-up along one or more timescales.

Within this background, for IV analysis we used a Poisson regression modelling framework with an offset for the log of survival time post discharge up to 365 days. After that, sensitivity analyses for the primary and secondary outcomes were repeated in a similar fashion as was done for the primary analysis using the IPCW-adjusted Cox models, but in this case, including the calendar year as the instrument of $P2Y_{12}$ inhibitor therapy to behave like a natural randomization of patients. Compared with standard survival modeling, IV analysis may derive less biased estimates of treatment effects in the setting that unmeasured cofounders affect both the decision to treat and the occurrence of outcome. As a result, the exponentiated coefficients (i.e., the incidence rates ratios, equivalent to the hazards ratios) with their 95% CIs were reported for both the primary and secondary outcomes (**Table S5**). Finally, the pre-specified assumptions were explored to determine the validity and robustness of the IV approach. Accordingly, the calendar day satisfied the three assumptions needed to be satisfied: (1) the IV had a higher strength of association with the exposure (**Figure S1**); (2) the validity of IV was examined by calculation of the standardized differences of baseline covariables that reflect known patient characteristics and procedural details according to the calendar year. Thereafter, the instrument was not significantly associated with measured confounders (**Table S6**); (3) nor with the outcomes (**Table S7**).

2.5.5 Intention-to-treat Analysis

We performed sensitivity analysis for the primary and secondary outcomes simulating the ITT approach used in previous studies. Such type of analysis does not account for treatment nonadherence, so follow-up is not censored at the time of discontinuing or switching the $P2Y_{12}$ inhibitor. Analysis of major bleeding according to the ITT principle revealed that those patients initially discharged on ticagrelor who switched to clopidogrel prior to the occurrence of a major bleeding complication, were erroneously classified with respect to exposure (exposure misclassification bias). Therefore, this caused the incidence of major bleeding to be falsely higher for ticagrelor- than clopidogrel-treated patients. Likewise, ITT analysis also resulted in unrealistic estimates towards the null with regards to ischemic outcomes (MACE), in full agreement with the results of previous studies based on this assumption for exposure (**Table S8**).

Using the ITT approach versus on-treatment analysis resulted in differential exposure misclassification bias in favor of ticagrelor over clopidogrel. Consequently, clinical outcomes that were misclassified as having occurred while on ticagrelor treatment were distributed as follows:

- Seven major bleeding events
- Five MACEs
- Two deaths (both were vascular deaths in origin)
- Nine myocardial infarctions
- One ischemic stroke *
- One urgent target lesion revascularization
- One definite stent thrombosis
- Two vascular deaths
- Ten NACEs events

* One ischaemic stroke was misclassified as having occurred on clopidogrel treatment, when actually the patient was receiving ticagrelor.

The vast majority of errors in classification of exposure status in relation to the occurrence of the study outcomes were observed in the ticagrelor-treated patients, when the event of interest preceded a strategy of de-escalation from ticagrelor to clopidogrel. This change in the exposure status in relation to the initial $P2Y_{12}$ inhibitor prescribed at hospital discharge—in which ITT analysis is based on—was not captured under the ITT assumption. Accordingly, given that de-escalation from ticagrelor to clopidogrel was the most frequent switching scenario, both in the current and other recent real-world studies, it would be expected that ITT analysis of observational data will result in higher rates of exposure misclassification for ticagrelor than clopidogrel.

Consequently, in the current study, the differential exposure misclassification introduced when the ITT principle was assumed for modelling the relationship between $P2Y_{12}$ inhibitor therapy and outcomes, ultimately resulted in a no significant effect on MACE reduction, and an unrealistic increased risk of major bleeding for ticagrelor compared with clopidogrel.

3 Supplementary Figures and Tables

3.1 Supplementary Tables

Table S1. Duration of DAPT according to the P2Y12 inhibitor-adherence pattern

	P2Y12 inhibitor treatment *			
**P2Y12 inhibitor discontinuation pattern/ DAPT duration †**	**Ticagrelor**	**Clopidogrel**	**Total**	**P value**
**Any discontinuation**	**73 (7.2)**	**120 (11.3)**	**193 (9.3)**	**0.001**
DAPT duration, days	302 (105)<br>362 (254─360)	279 (119)<br>350 (189─365)	288 (114)<br>352 (213─366)	0.180
**Permanent Discontinuation**	**50 (5.0)**	**100 (9.5)**	**150 (7.2)**	**< 0.001**
DAPT duration, days	221 (102)<br>219 (140─332)	204 (117)<br>210 (91─322)	209 (113)<br>214 (101─326)	0.350
**Brief / Temporary Interruption**	23 (2.2)	20 (2.0)	43 (2.1)	0.890
DAPT duration, days	342 (75)<br>366 (365─378)	327 (91)<br>365 (360─379)	334 (82)<br>365 (364─367)	0.570

Bold font indicates statistical significance at P <0.05.
Data are expressed as n (%), mean (SD), or median (IQR), as appropriate.
(%) are Kaplan-Meier discontinuation rates
DAPT discontinuation is defined as withdrawal of the $P2Y_{12}$ inhibitor
† Total duration of DAPT expressed in days from the date of index admission until the date of last exposure, death, or censoring, whichever occurred first.
* $P2Y_{12}$ inhibitor exposure status was ascertained according to the on-treatment principle. That is the $P2Y_{12}$ inhibitor actually received at the time of drug discontinuation, death, or censoring.

Abbreviations: DAPT, dual antiplatelet therapy, IQR, inter-quartile range; SD standard deviation

Table S2. Timing of permanent DAPT discontinuation

	P2Y12 inhibitor treatment *			
	**Ticagrelor (n= 1012)**	**Clopidogrel (n= 1058)**	**Total (n= 2070)**	***P* value**
**Permanent Discontinuation/ Time Interval †**	**50 (5.0)**	**100 (9.5)**	**150 (7.2)**	**< 0.001**
< 30 days	2 (4.0)	6 (6.0)	8 (5.3)	0.410‡
30-90 days	5 (10.0)	19 (19.0)	24 (16.0)	
90-150 days	6 (12.0)	12 (12.0)	18 (12.0)	
150-210 days	11 (22.0)	12 (12.0)	23 (15.3)	
210-270 days	7 (14.0)	12 (12.0)	19 (12.7)	
270-365 days	19 (38.0)	39 (39.0)	58 (38.7)	

Bold font indicates statistical significance
Data are n (Kaplan-Meier discontinuation rates, %)
DAPT discontinuation is defined as the withdrawal of the $P2Y_{12}$ inhibitor
* $P2Y_{12}$ inhibitor exposure status was ascertained according to the on-treatment principle. That is the $P2Y_{12}$ inhibitor actually received at the time of drug discontinuation, death, or censoring.
† Time intervals expressed in days from the date of index admission
‡ Cochran–Armitage test for linear trend across time intervals.

Table S3. Reasons for DAPT discontinuation

	P2Y12 inhibitor treatment *			
**DAPT discontinuation**	**Ticagrelor (n= 1012)**	**Clopidogrel (n= 1058)**	**Total (n= 2070)**	**P value**
**Any discontinuation**	**73 (7.2)**	**120 (11.3)**	**193 (9.3)**	**0.001**
Physician-guided	36 (49.3)	67 (56.0)	103 (53.3)	**0.040**†
Bleeding event	14 (19.2)	29 (24.0)	43 (22.3)	
Invasive procedure	14 (19.2)	20 (16.6)	34 (16.7)	
Side effect (dyspnea)	2 (2.7)	-	2 (1.0)	
Non-compliance	**7 (9.6)**	**4 (3.4)**	**11 (5.7)**	
**Permanent discontinuation**	**50 (5.0)**	**100 (9.5)**	**150 (7.2)**	**< 0.001**
Physician-guided	27 (54.0)	66 (66.0)	93 (62.0)	**0.020**[b]
Bleeding event	12 (24.0)	22 (22.0)	34 (22.5)	
Invasive procedure	6 (12.0)	10 (10.0)	16 (10.5)	
Side effect (dyspnea)	1 (2.0)	-	1 (0.7)	
Non-compliance	**4 (8.0)**	**2 (2.0)**	**6 (0.4)**	
**Transient discontinuation**	23 (2.2)	20 (2.0)	43 (2.1)	0.890
Physician-guided	9 (39.0)	1 (5.0)	10 (23.2)	**0.030**[b]
Bleeding event	2 (8.7)	7 (35.0)	9 (21.0)	
Invasive procedure	8 (35.0)	10 (50.0)	18 (42.0)	
Side effect (dyspnea)	1 (4.3)	-	1 (2.2)	
Non-compliance	3 (13.0)	2 (10.0)	5 (11.6)	

Bold font indicates statistical significance
Data are n (%)
DAPT discontinuation is defined as the withdrawal of the $P2Y_{12}$ inhibitor
* $P2Y_{12}$ inhibitor exposure was ascertained according to the on-treatment principle.
† Cochran–Armitage test for linear trend across categories

Table S4. Results of the Propensity Score Matching Analysis

	Cumulative incidence, *no. events (%)* *			adjusted Model †		
	Total (n= 1,602)	**Ticagrelor (n= 801)**	**Clopidogrel (n= 801)**	**Hazard Ratio**	**95%CI**	**P value**
**Primary Outcome**						
**BARC type 3 or 5 bleeding**	79 (5.20)	50 (6.54)	29 (3.80)	1.44	0.99 – 2.08	0.05
**Secondary Outcomes**						
**MACE**	142 (9.10)	55 (7.20)	87 (10.5)	0.62	0.39 – 0.98	**0.04**
All-cause death	74 (4.60)	29 (3.73)	45 (5.47)	0.71	0.51 – 0.99	**0.04**
Non-fatal myocardial infarction	62 (4.20)	21 (3.05)	41 (5.27)	0.56	0.33 – 0.93	**0.02**
Non-fatal stroke	17 (1.10)	5 (0.65)	12 (1.60)	0.55	0.29 – 1.04	0.06
Urgent target lesion revascularization	52 (3.57)	24 (3.48)	28 (3.65)	0.86	0.45 – 1.67	0.67
**Stent thrombosis (definite/probable)**	45 (2.90)	18 (2.40)	27 (3.40)	0.66	0.45 – 0.95	**0.02**
**Vascular death**	41 (2.58)	19 (2.45)	22 (2.70)	0.78	0.63 – 0.98	**0.03**
**NACE**	199 (12.5)	89 (11.2)	110 (13.7)	0.76	0.60 – 0.95	**0.01**

Boldface font denotes statistical significance set at P < 0.05

*(%) are weighted Kaplan-Meier estimates or cumulative incidence functions at 1 year, as appropriate
† IPCW-covariate fully adjusted Cox or Fine-Gray regression models with individual hospitals entered as a random-effects variable

BARC, Bleeding Academic Research Consortium; CI, confidence interval; IPCW, inverse propensity of censoring weighting; MACE, major adverse cardiac event; NACE, net adverse clinical event.

Table S5. Results of Instrumental Variable Analysis

	Cumulative incidence, *no. events (%)* *			adjusted Model †		
	**Total (n= 2,070)**	**Ticagrelor (n= 1,035)**	**Clopidogrel (n= 1,035)**	**IRR (HR)**	**95%CI**	**P value**
**Primary Outcome**						
**BARC type 3 or 5 bleeding**	106 (5.36)	55 (5.60)	51 (5.20)	1.24	0.81 – 1.90	0.320
**Secondary Outcomes**						
**MACE**	198 (10.2)	63 (6.35)	135 (13.7)	0.59	0.37 – 0.95	**0.030**
All-cause death	105 (5.08)	30 (2.95)	75 (7.13)	0.43	0.25 – 0.76	**0.004**
Non-fatal myocardial infarction	82 (4.22)	24 (2.48)	59 (5.97)	0.49	0.29 – 0.83	**0.008**
Non-fatal stroke	23 (1.15)	7 (0.70)	16 (1.64)	0.63	0.39 – 1.01	0.060
Urgent target lesion revascularization	66 (3.30)	29 (3.02)	37 (3.63)	0.88	0.52 – 1.51	0.660
**Stent thrombosis (definite/probable)**	59 (2.95)	21 (2.10)	38 (3.70)	0.57	0.35 – 0.95	**0.030**
**Vascular death**	55 (2.70)	19 (1.87)	36 (3.50)	0.51	0.38 – 0.70	**0.001**
**NACE**	274 (13.3)	108 (10.6)	166 (15.9)	0.81	0.66 – 0.98	**0.044**

Boldface font denotes statistical significance

* (%) are weighted Kaplan-Meier estimates or cumulative incidence functions at 1 year, as appropriate

† Instrumental variable piecewise Poisson regression model with covariate adjustments and individual hospitals entered as a random-effects variable (IRR are the exponentiated coefficients of piecewise Poisson models, which are analogous to hazard ratios in Cox regression assuming constant baseline hazards)

BARC, Bleeding Academic Research Consortium; CI, confidence interval; HR; hazard ratio; IRR, incidence rate ratio; MACE, major adverse cardiac event; NACE, net adverse clinical event

Table S6. Baseline data for patients discharged on clopidogrel or ticagrelor by calendar year

	2015 (n = 351)	2016 (n = 575)	2017 (n = 671)	2018/19 (n = 473)	SMD
Age, years	63.5 (12.0)	63 (12.4)	63.0 (12.1)	65.0 (13.0)	-0.08
Sex, Male	259 (74.0)	430 (75.0)	493 (73.5)	335 (71.0)	0.02
Body mass index, kg/m^2	27.7 (4.0)	27.6 (4.2)	27.8 (4.4)	27.6 (4.0)	-0.01
Past medical history					
Current smoker	144 (41.0)	251 (43.8)	289 (42.0)	159 (40.0)	-0.03
Hypertension	201 (57.3)	312 (54.3)	374 (55.7)	279 (59.0)	-0.03
Diabetes mellitus	208 (31.0)	160 (28.2)	203 (30.0)	172 (36.2)	-0.06
Hyperlipidemia	158 (45.1)	261 (45.5)	297 (44.3)	221 (46.5)	0.00
Peripheral arterial disease	23 (6.5)	26 (4.5)	43 (6.4)	23 (5.0)	-0.01
Chronic obstructive pulmonary disease	28 (8.2)	27 (4.7)	43 (6.4)	20 (4.2)	0.05
Chronic kidney disease	18 (5.2)	32 (5.5)	44 (6.6)	41 (8.7)	-0.08
Dialysis	6 (1.7)	12 (2.2)	10 (1.5)	8 (1.7)	0.06
History of atrial fibrillation	5 (1.4)	17 (3.1)	18 (2.7)	7 (1.5)	0.01
Previous myocardial infarction	54 (15.4)	65 (11.3)	102 (15.2)	83 (17.5)	-0.06
Previous PCI	55 (15.6)	76 (13.4)	90 (13.5)	81 (17.3)	-0.03
Previous CABG	5 (1.5)	14 (2.5)	12 (2.1)	8 (1.7)	0.01
History of stroke	29 (8.3)	36 (6.3)	50 (7.5)	38 (8.0)	0.01
Heart failure, (NYHA class > III)	4 (1.1)	5 (1.2)	18 (2.7)	10 (2.0)	-0.08
Previous major bleeding	7 (2.2)	12 (2.1)	20 (3.0)	19 (4.0)	-0.08
History of anaemia	12 (3.5)	13 (2.3)	25 (3.7)	24 (5.0)	-0.04
Prior cancer diagnosis *	8 (2.3)	7 (1.5)	11 (1.5)	13 (2.5)	-0.02
Clinical presentation					
Non-ST-segment elevation ACS	121 (34.5)	206 (36.2)	263 (39.2)	189 (40.0)	-0.06
Non–ST-segment elevation MI	109 (31.5)	174 (30.5)	230 (34.3)	173 (36.5)	-0.02
Unstable angina	12 (3.5)	32 (5.5)	33 (5.0)	16 (3.5)	0.01
ST-segment elevation MI	230 (65.5)	369 (64.3)	408 (61.5)	284 (60.3)	0.06
Killip class ≥ 2	45 (13.5)	72 (12.5)	73 (11.5)	65 (13.7)	0.01
CRUSADE score	25 (15, 38)	24 (14, 37)	24 (13, 37)	27 (15, 40)	-0.06
GRACE score	136 (114, 161)	136 (112, 161)	133 (110, 156)	141 (120, 169)	-0.03
Creatinine clearance, ml/min/1.73 m^2	81 (58, 108)	86 (61, 112)	88 (62, 110)	81 (56, 108)	-0.04
CrCl< 30	16 (4.6)	28 (5.1)	29 (4.3)	27 (5.6)	0.00
LVEF at discharge, %	51 (11.2)	52 (10.6)	52 (10.4)	52 (11.4)	-0.01
Procedural characteristics					
Radial artery approach	164 (46.7)	320 (55.7)	502 (74.8)	377 (79.7)	-0.20
Multivessel disease	145 (41.3)	246 (42,8)	308 (45.9)	232 (49.0)	-0.09
Chronic total occlusion	24 (6.8)	40 (7.8)	52 (7.7)	42 (8.9)	-0.05
Complete revascularization †	97 (67.0)	134 (54.5)	172 (55.8)	134 (57.8)	0.06
Glycoprotein IIb/IIIa inhibitors	45 (12.8)	46 (8.0)	79 (11.8)	60 (12.7)	-0.07
Reperfusion therapy, STEMI					
Primary PCI	186 (81.0)	299 (81.0)	346 (84.5)	252 (88.7)	-0.09
Pharmacoinvasive strategy	44 (18.7)	70 (19.0)	63 (15.4)	62 (11.3)	0.08
Management strategy, NSTE-ACS					
Invasive	118 (97.5)	202 (98.1)	261 (99.2)	187 (98.9)	-0.08
Conservative	3 (2.5)	4 (1.9)	2 (0.8)	2 (1.1)	0.07
Reperfusion strategy, all-comers					
PCI, any	317 (90.3)	513 (89.2)	606 (90.3)	421 (89.0)	0.03
Drug-eluting stent	244 (69.5)	416 (72.3)	552 (82.3)	396 (83.7)	-0.29
CABG	6 (1.7)	13 (2.3)	11 (1.6)	11 (2.3)	-0.01
Medical treatment	28 (8.0)	49 (8.5)	54 (8.0)	41 (8.7)	0.00

Table S6 (continued)

	2015 (n = 351)	2016 (n = 575)	2017 (n = 671)	2018/19 (n = 473)	SMD
Medication at discharge/Adherence					
β-Blocker	300 (85.5)	495 (86.0)	582 (86.5)	412 (87.1)	-0.03
Statin	340 (97.0)	563 (98.0)	648 (96.6)	449 (95.0)	0.01
RAAS blocker	323 (97.0)	507 (88.2)	597 (89.0)	422 (89.0)	0.01
Proton-pump inhibitor / H2-receptor blocker	268 (76.4)	427 (74.3)	526 (78.5)	370 (78.2)	-0.06
$P2Y_{12}$ inhibitor switching after discharge	14 (4.0)	27 (4.7)	33 (5.0)	28 (5.9)	-0.05
Time-to-switch, days	38 (74, 174)	25 (4, 107)	31 (7, 101)	100 (33, 179)	-0.08
Duration of DAPT, days	342 (71)	348 (68)	347 (74)	348 (74)	-0.01
DAPT discontinuation	45 (12.8)	49 (8.5)	60 (8.9)	39 (8.2)	0.06
Medication possession ratio (1-year), %	0.74 (0.29)	0.75 (0.28)	0.80 (0.25)	0.72 (0.26)	0.00

All data are shown as n (%), mean (SD), or as median (inter-quartile range)
* Diagnosis of cancer more than 3 years before the index ACS admission
† For patients with multivessel coronary artery disease
SMDs <10% indicate adequate balance between matched samples.

Abbreviations: SMD, standardized mean differences; rest of abbreviations as in Figure S4

Table S7. Primary and secondary outcomes according to calendar day

	**Cumulative incidence,** ***no. events (%)*** *				***P* value** †
	**2015 (n = 351)**	**2016 (n = 575)**	**2017 (n = 671)**	**2018/19 (n = 473)**	
**Primary Outcome**					
**BARC type 3 or 5 bleeding**	19 (5.4)	27 (4.7)	25 (3.7)	35 (7.4)	0.07
**Secondary Outcomes**					
**MACE**	32 (9.1)	50 (8.7)	65 (9.7)	51 (10.9)	0.66
All-cause death	16 (4.5)	29 (5.0)	39 (5.8)	21 (4.4)	0.72
Non-fatal myocardial infarction	12 (3.6)	23 (4.2)	23 (4.2)	20 (4.8)	0.92
Non-fatal stroke	4 (1.2)	6 (1.1)	3 (0.5)	10 (2.2)	0.10
Urgent target lesion revascularization	10 (3.1)	18 (3.3)	20 (3.2)	18 (4.3)	0.77
**Stent thrombosis (definite/probable)**	10 (2.9)	10 (1.8)	24 (3.7)	15 (3.3)	0.25
**Vascular death**	8 (2.3)	15 (2.6)	17 (2.5)	15 (3.2)	0.86
**NACE**	45 (12.8)	69 (12.0)	78 (11.7)	81 (17.2)	0.07

* (%) are censoring weighted Kaplan-Meier estimates, or cumulative incidence functions at 1 year
† *P* values derived from weighted log-rank test or Gray´s test, as appropriate.

BARC, Bleeding Academic Research Consortium; CI, confidence interval; MACE, major adverse cardiac event; NACE, net adverse clinical event

Table S8. Intention-to-treat analysis of 1-year outcomes in (A) the overall study cohort and (B) the propensity-score matched patients with acute coronary syndrome treated with either ticagrelor or clopidogrel

A. Overall cohort

	Cumulative incidence, *no. events (%)* *			Fully adjusted Model †		
	Total (n= 2070)	Ticagrelor (n= 1035)	Clopidogrel (n= 1035)	Hazard Ratio	95%CI	P value
**Primary Outcome**						
**BARC type 3 or 5 bleeding**	105 (5.30)	62 (6.58)	43 (3.80)	1.64	1.22 – 2.21	**0.001**
**Secondary Outcomes**						
**MACE**	198 (9.60)	68 (6.60)	130 (12.52)	0.72	0.50 – 1.04	0.080
All-cause death	105 (5.05)	32 (3.10)	73 (7.06)	0.65	0.41 – 1.02	0.060
Non-fatal myocardial infarction	83 (4.33)	33 (3.44)	50 (5.23)	0.68	0.39 – 1.04	0.070
Non-fatal stroke	23 (1.13)	6 (0.60)	17 (1.73)	0.55	0.30 – 1.03	0.060
Urgent target lesion revascularization	66 (3.46)	30 (3.13)	36 (3.70)	0.87	0.53 – 1.45	0.610
**Stent thrombosis (definite/probable)**	58 (2.82)	22 (2.16)	36 (3.47)	0.71	0.49 – 1.04	0.080
**Vascular death**	55 (2.68)	21 (2.04)	34 (3.34)	0.78	0.51 – 1.21	0.280
**NACE**	274 (13.25)	118 (11.37)	156 (15.14)	0.71	0.49 – 1.03	0.070

B. Propensity-score matched sample

	Cumulative incidence, *no. events (%)* *			Fully adjusted Model †		
	Total (n= 1602)	Ticagrelor (n= 801)	Clopidogrel (n= 801)	Hazard Ratio	95%CI	P value
**Primary Outcome**						
**BARC type 3 or 5 bleeding**	79 (5.10)	50 (6.50)	29 (3.72)	1.95	1.27 – 2.98	**0.002**
**Secondary Outcomes**						
**MACE**	142 (8.85)	59 (7.42)	83 (10.31)	0.68	0.73 – 1.09	0.110
All-cause death	74 (4.62)	30 (3.77)	44 (5.46)	0.71	0.42 – 1.25	0.220
Non-fatal myocardial infarction	64 (4.33)	26 (3.60)	38 (5.05)	0.73	0.40 – 1.33	0.310
Non-fatal stroke	17 (1.15)	4 (0.50)	13 (1.63)	0.50	0.25 – 1.03	0.050
Urgent target lesion revascularization	52 (3.51)	25 (3.40)	27 (3.60)	0.84	0.41 – 1.73	0.640
**Stent thrombosis (definite/probable)**	29 (1.95)	14 (1.80)	15 (1.95)	0.95	0.46 – 1.96	0.900
**Vascular death**	41 (2.58)	20 (2.50)	21 (2.65)	0.89	0.44 – 1.81	0.760
**NACE**	199 (12.45)	89 (11.19)	110 (13.65)	0.74	0.49 – 1.14	0.170

Boldface font indicates statistically significance ($P< 0.05$)

* (%) are crude Kaplan-Meier estimates or cumulative incidence functions at 1 year, as appropriate

† Fully adjusted Cox or Fine-Gray regression models with robust variance estimators (individual hospitals entered as a random-effects variable)

Abbreviations: BARC, Bleeding Academic Research Consortium; CI, confidence interval; MACE, major adverse cardiac event; NACE, net adverse clinical event

3.2 Supplementary Figures

(A)

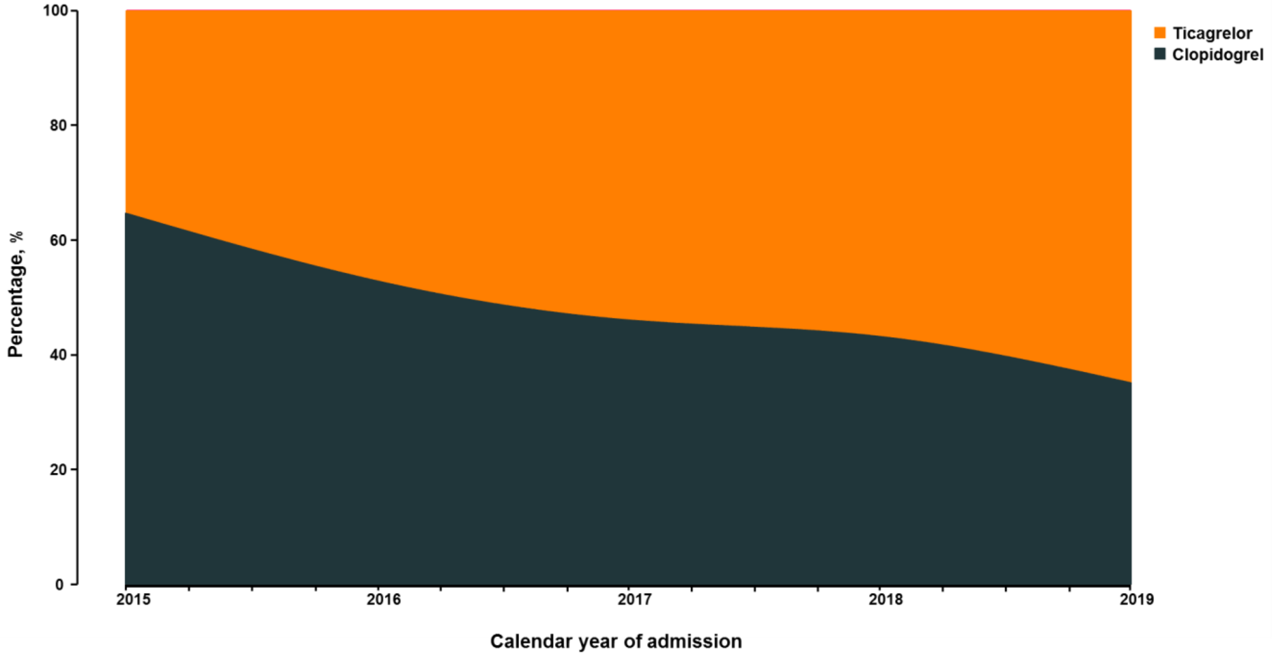

Panel A shows temporal trends of ticagrelor and clopidogrel use in patients with acute coronary syndrome in the CREA-ARIAM Andalucía registry between March 2015 and April 2019.
Cochran-Armitage test for linear trend over time, *P* for trend < 0.001

(B)

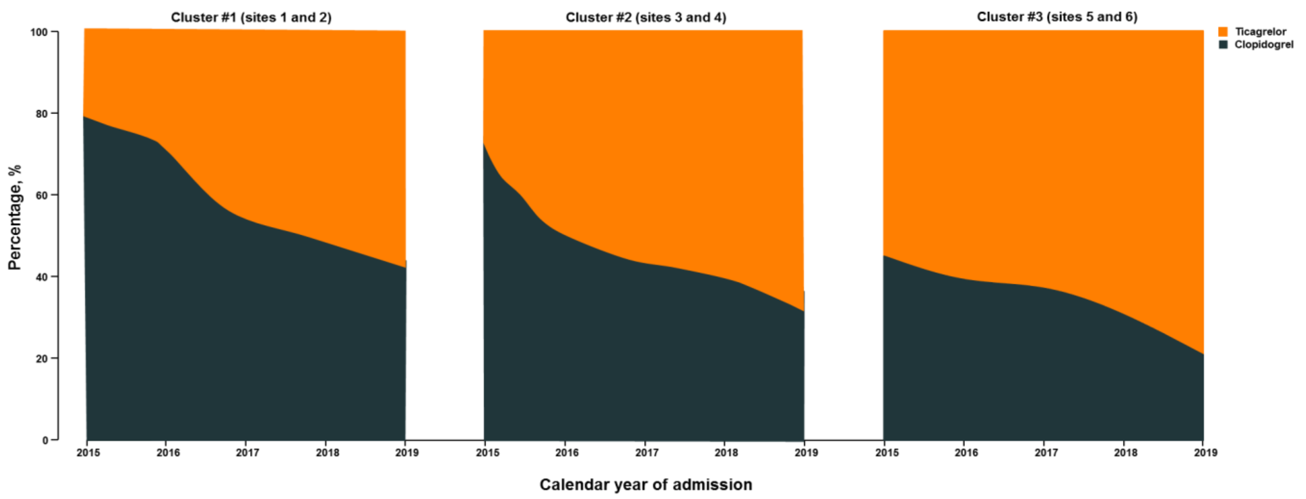

Panel B depicts the temporal trend of use of ticagrelor and clopidogrel across the six participating centres grouped into three clusters, according to the geographical region and prescribing pattern of $P2Y_{12}$ inhibitors at hospital discharge. Each cluster consisted of two nearby hospitals with similar pattern of $P2Y_{12}$ inhibitors use over the study period.
Cochran-Armitage test for linear trend in proportions across the clusters over the study period; *P = 0.08*

Supplementary Figure 1. Temporal trends of ticagrelor and clopidogrel use during the study period in (A) the entire cohort and (B) stratified by participating centers grouped into clusters

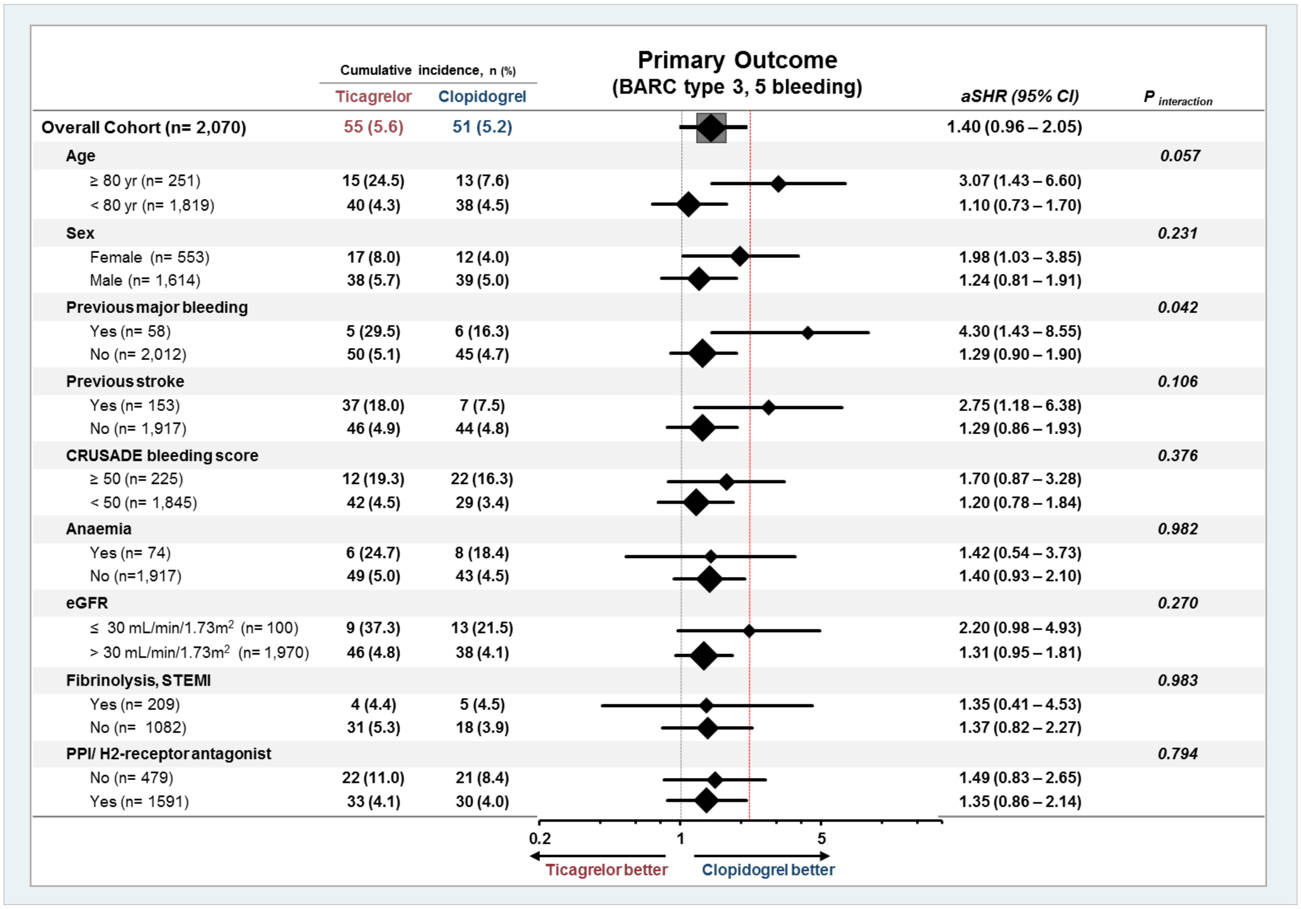

Supplementary Figure 2. Subgroup analysis of BARC type 3 or 5 bleeding

The forest plot depicts the adjusted subdistribution *hazard ratios* for the primary endpoint stratified by $P2Y_{12}$ inhibitor across pre-specified subgroups.

P-values for formal tests of interaction are not adjusted for multiple comparisons.

Diamonds and continuous horizontal lines in black represent the *aSHRs* (for ticagrelor *vs* clopidogrel) and their 95% CIs, respectively. The diamond area is proportional to the number of subjects in each subgroup.

The vertical dashed line in red set at 2.1 represents the estimated threshold for the upper bound of the two-sided 95% CI of the *aSHR* to reject the null hypothesis that clopidogrel is safer than ticagrelor in terms of major bleeding risk.

Abbreviations: aSHR, adjusted subdistribution hazard ratio; ACS, acute coronary syndrome; CI, confidence interval; CRUSADE, Can Rapid risk stratification of Unstable angina patients Suppress ADverse outcomes with Early implementation of the ACC/AHA guidelines; BARC, Bleeding Academic Research Consortium; eGFR, estimated glomerular filtration rate; GRACE, Global Registry of Acute Coronary Events; PPI, proton pump inhibitor; STEMI, ST-segment elevation MI

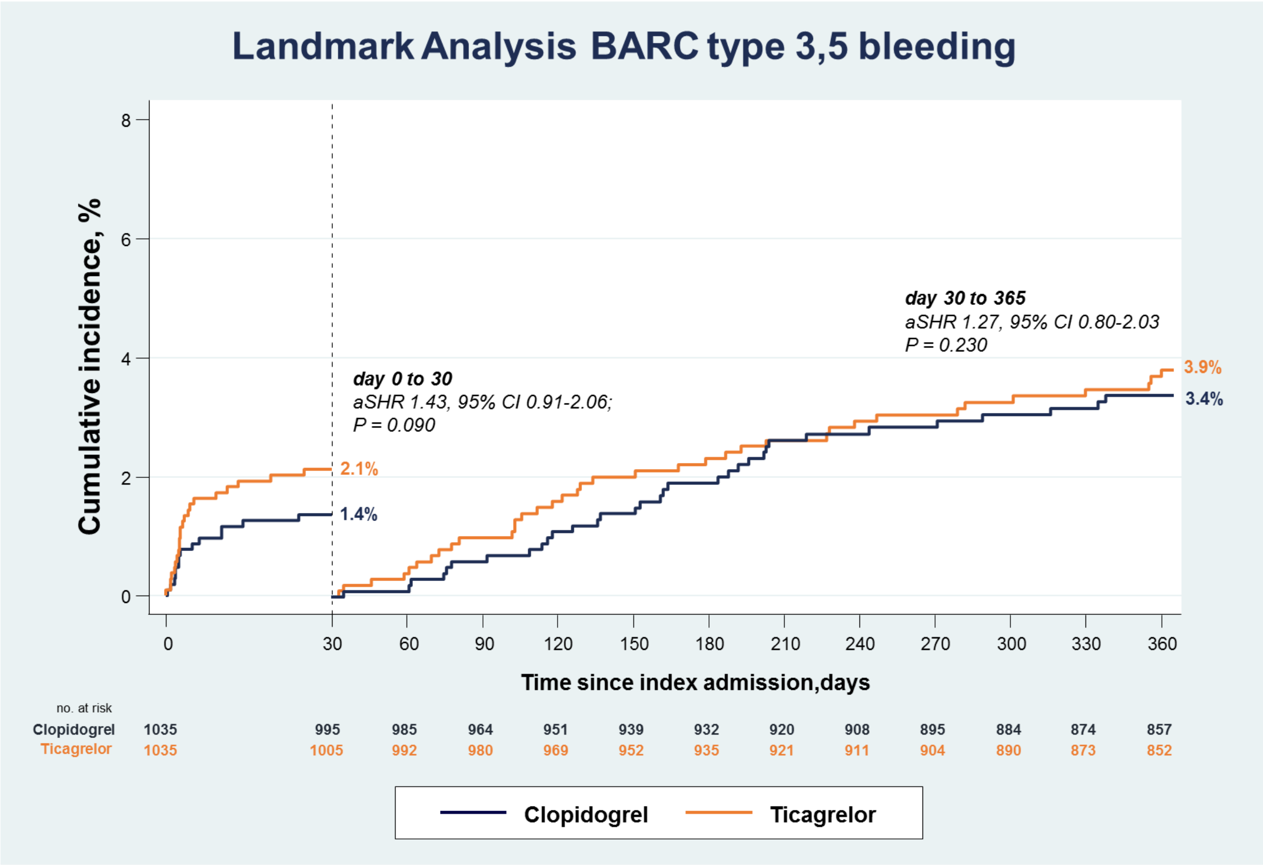

Supplementary Figure 3. Landmark analysis of major bleeding events at 30 days

30-day landmark analysis of major bleeding events

The curves represent the IPCW-adjusted cumulative incidence functions of major bleeding events for ticagrelor and clopidogrel, in two separate period of analysis: first analysis from index admission to day 30, and the second analysis between 30 and 365 days. Neither the early analysis within 30 days after admission, nor that from day 30 onwards showed signals of harm for ticagrelor compared to clopidogrel. Both analysis fulfilled the primary study hypothesis that ticagrelor would be at least as safe as clopidogrel in terms of bleeding risk (i.e., the upper bound of the 95%CIs for the adjusted *SHR (aSHR)* derived from competing risks regression models was below 2.10) as shown: upper bound of the 95% CI for the *aSHR* 2.06 and 2.03, for models evaluating the relationship between bleeding and exposures from day 0 to day 30, and from day 30 to day 365, respectively. In addition, the analysis suggested that the more pronounced, though not statistically significant risk of bleeding for ticagrelor compared with clopidogrel within 30 days after index admission, was progressively less evident over time.

Abbreviations: aSHR, adjusted subhazard ratio; BARC, Bleeding Academic Research Consortium; CI, confidence interval

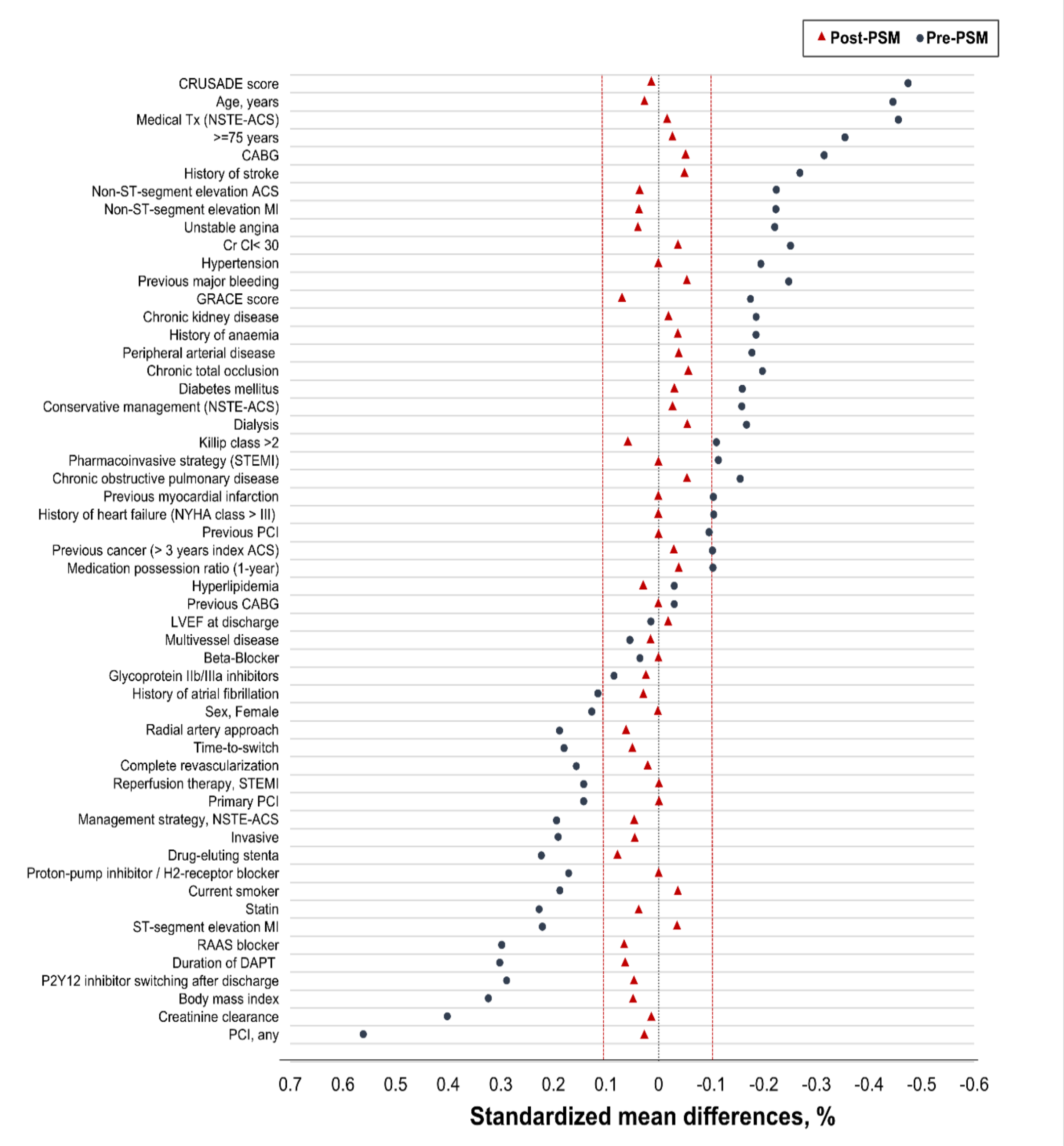

Supplementary Figure 4. Standardized mean difference plot for covariates before (overall cohort) and after matching (matched sample)

Standardized mean differences (SMDs) for baseline covariates comparing subjects treated with ticagrelor and clopidogrel in the original (Pre-PSM); and the matched sample (Post-PSM). The dotted vertical lines in red indicate the cutoff for mean standardized difference (0.1), indicating an acceptable balance of covariate distribution between treatment groups after matching. SMDs for all covariates after matching are well within the pre-specified margin.

Abbreviations: ACS, acute coronary syndrome; CRUSADE, Can Rapid risk stratification of Unstable angina patients Suppress ADverse outcomes with Early implementation of the ACC/AHA guidelines; BARC, Bleeding Academic Research Consortium; CrCl, creatinine clearance; DAPT, dual antiplatelet therapy; GRACE, Global Registry of Acute Coronary Events; LVEF, left ventricular ejection fraction; MI, myocardial infarction; NSTE-ACS, Non-ST-segment elevation ACS; PCI, percutaneous coronary intervention; PSM, propensity score matching; RAAS, renin-angiotensin-aldosterone system; STEMI, ST-segment elevation MI
